# Hypoxia-reprogramed megamitochondrion contacts and engulfs lysosome to mediate mitochondrial self-digestion

**DOI:** 10.1038/s41467-023-39811-9

**Published:** 2023-07-11

**Authors:** Tianshu Hao, Jianglong Yu, Zhida Wu, Jie Jiang, Longlong Gong, Bingjun Wang, Hanze Guo, Huabin Zhao, Bin Lu, Simone Engelender, He He, Zhiyin Song

**Affiliations:** 1 College of Life Sciences, Taikang center for life and medical sciences, Frontier Science Center for Immunology and Metabolism, Department of Anesthesiology, Renmin Hospital of Wuhan University, Wuhan University, Wuhan, 430072 Hubei China; 2 Department of Biochemistry and Molecular Biology, School of Basic Medical Sciences, Hengyang Medical School, University of South China, Hengyang, 421001 Hunan China; 3 Department of Biochemistry, Rappaport Faculty of Medicine, Technion-Israel Institute of Technology, Haifa, Israel

**Keywords:** Mitochondria, Lysosomes, Lysosomes, Hypoxia

## Abstract

Mitochondria are the key organelles for sensing oxygen, which is consumed by oxidative phosphorylation to generate ATP. Lysosomes contain hydrolytic enzymes that degrade misfolded proteins and damaged organelles to maintain cellular homeostasis. Mitochondria physically and functionally interact with lysosomes to regulate cellular metabolism. However, the mode and biological functions of mitochondria-lysosome communication remain largely unknown. Here, we show that hypoxia remodels normal tubular mitochondria into megamitochondria by inducing broad inter-mitochondria contacts and subsequent fusion. Importantly, under hypoxia, mitochondria-lysosome contacts are promoted, and certain lysosomes are engulfed by megamitochondria, in a process we term megamitochondria engulfing lysosome (MMEL). Both megamitochondria and mature lysosomes are required for MMEL. Moreover, the STX17-SNAP29-VAMP7 complex contributes to mitochondria-lysosome contacts and MMEL under hypoxia. Intriguingly, MMEL mediates a mode of mitochondrial degradation, which we termed mitochondrial self-digestion (MSD). Moreover, MSD increases mitochondrial ROS production. Our results reveal a mode of crosstalk between mitochondria and lysosomes and uncover an additional pathway for mitochondrial degradation.

## Abstract (web summary)

Several organelle membranes make contact in the cell, with many contacts being spatially segregated sites dedicated to specific functions. Here, Hao et al. show that hypoxia increases mitochondria-lysosome contacts, leading to engulfment and degradation of the mitochondria.

## Introduction

Mitochondrion is a double membrane organelle whose primary function is to generate ATP by oxidative phosphorylation in the cell^1,2^. Mitochondria are continuously fusing and dividing to control their size, number, morphology, and functions. Mitofusins (*MFN1* and *MFN2*) and *OPA1*/*mgm1* are required for mitochondrial outer and inner membrane fusion, respectively^3^. In contrast, DRP1 is an important mitochondrial fission factor that is recruited to the mitochondrial outer membrane by *MFF*, *FIS1*, *MID49/51* for mediating mitochondrial fission^3,4^. Inhibition of mitochondrial fusion leads to the mitochondrial fragmentation, and lack of mitochondrial fission leads to mitochondrial elongation^4^. In addition, depletion of the MICOS complex leads to the formation of large spherical mitochondria (megamitochondria) due to the termination of mitochondrial fission and disorder of mitochondrial inner membrane organization^5,6^. Moreover, megamitochondria are frequently observed in aged cells and certain pathological cells and are associated with various human diseases^7–9^. However, the physiological functions of megamitochondria are poorly understood.

Lysosome is a single membrane-bound organelle that senses nutrient availability and initiates an adaptive response to maintain cellular homeostasis by digesting most macromolecules. Lysosomes contain over 60 hydrolytic enzymes capable of digesting almost all types of biological polymers including proteins, carbohydrates, nucleic acids, and lipids^10^. Lysosomes are also responsible for the degradation of various dysfunctional organelles, such as mitochondria. The damaged or dysfunctional mitochondria can be selectively engulfed by autophagosomes and delivered to lysosomes for degradation in a process known as mitophagy^11^.

Mitochondrial quality control is central to cellular metabolism. In addition to mitophagy by the autophagosome-lysosome system, mitochondrial quality also is regulated by a number of mitochondrial proteases. Mitochondria contain more than 40 proteases, including LONP1, PARL, Yme1L, AFG3L2, SPG7, OMA1, MPP, CLPP, CLPX, and HTRA2, which are responsible for the processing and degradation of mitochondrial proteins under certain conditions^12,13^. Mitochondrial proteases are also involved in mitophagy. Dysfunction of mitochondrial proteases leads to accumulation of unfolded or dysfunctional mitochondrial proteins that trigger mitochondrial unfolded protein response (UPR^mt^) and induce mitophagy^14^.

Mitochondrial and lysosomal activities appear to be tightly connected. Mitochondrial dysfunction leads to the impairment of lysosomes^15–17^. On the other hand, lysosome supports mitochondrial metabolism by regulating iron homeostasis;^18,19^ in contrast, dysfunctional lysosomes impair mitochondrial functions, leading to the accumulation of damaged mitochondria^11^. Further evidence for this interaction is that mitochondria and lysosome communicate physically and functionally^20–23^. In yeast, mitochondria are in physical contact with vacuoles (the lysosomal compartment of yeast) via vCLAMP^24^. In mammalian cells, lysosome can directly contact mitochondria to drive mitochondrial fission^23^. In addition, the Syntaxin17 (STX17)-SNAP29-VAMP7 SNARE complex mediates binding and fusion between MDV (mitochondrial-derived vesicles) and endolysosome^25^. The interplay between mitochondria and lysosomes is emerging as a critical determinant of cellular functions and associated diseases^11,26^, Nevertheless, the underlying molecular mechanisms of the interaction between mitochondria and lysosomes remain to be elucidated.

Hypoxia, a common physiological stress, directly activates hypoxia-inducible factor-1 (HIF-1), which is the major transcription factor regulating cellular responses to hypoxia. Under hypoxia, mitochondria act as oxygen sensors that relay signals to HIF-1 and contribute to the reprogramming of energy metabolism^27^. In addition, Higd-1a plays a role in cell survival by interacting with OPA1 under hypoxia^28^. Hypoxia can activate the mitophagy receptors FUNDC1, BNIP3, and BNIP3L to induce mitophagy via the autophagosome-lysosome pathway^29,30^. However, a large number of mitochondria are still present during both short and long hypoxia, suggesting that mitophagy does not degrade all dysfunctional mitochondria during hypoxia (or is not efficient enough to do so). Furthermore, it is unclear what function the remaining mitochondria have under hypoxia.

Here, we show that hypoxia reprograms normal tubular mitochondria into megamitochondria (large spherical mitochondria) that engulfs the lysosome, a process we now call megamitochondria engulfing lysosome (MMEL). MMEL and hypoxia-activated mitochondrial proteases contribute to mitochondrial protein degradation, which we call mitochondrial self-digestion (MSD). MSD promotes mitochondrial ROS production under hypoxia. Thus, we reveal a mode of contacts between mitochondria and lysosomes and uncover aa additional pathway for mitochondrial degradation.

## Results

### Hypoxia reshapes tubular mitochondria into megamitochondria

**Fig. 1:**
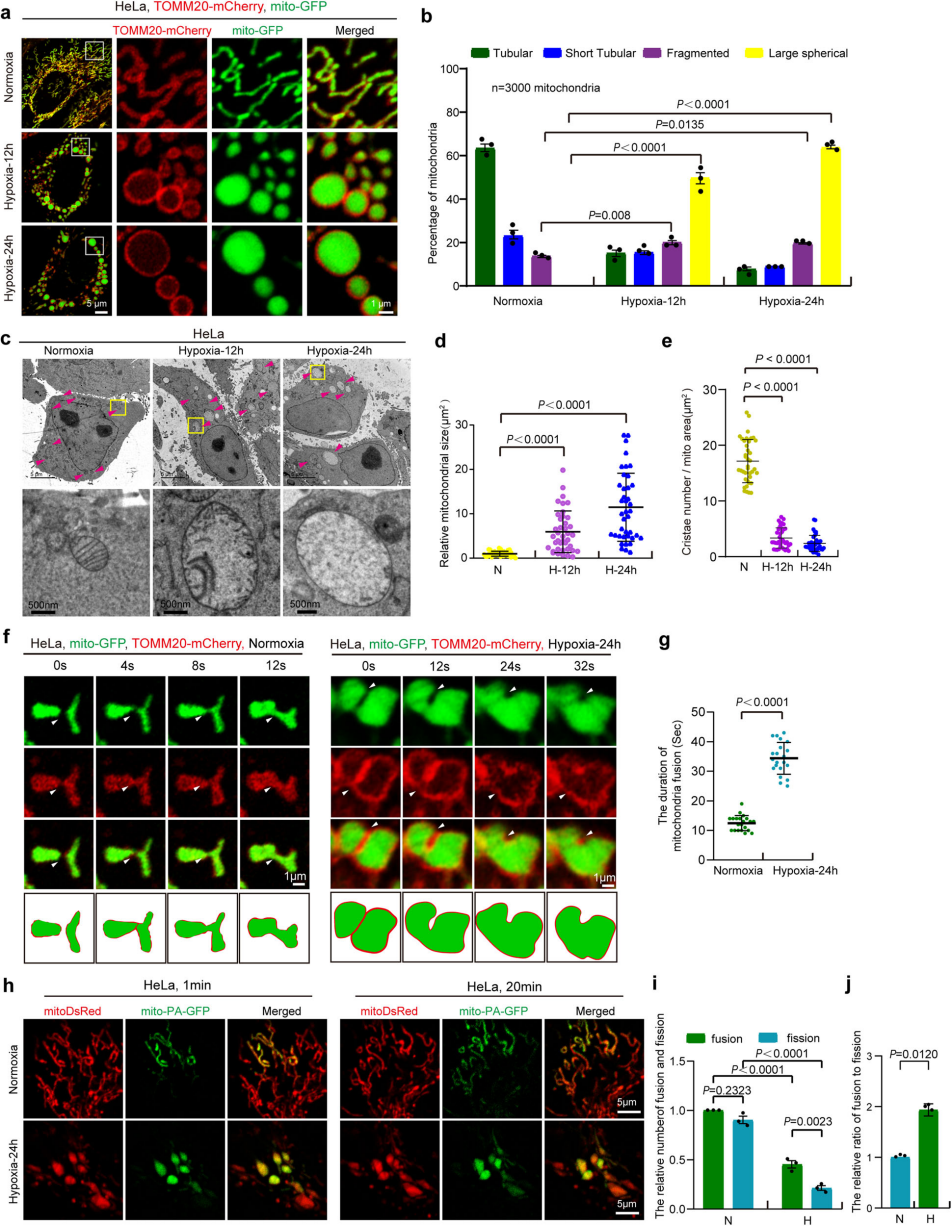
Hypoxia reshapes the normal tubular mitochondria to be megamitochondria. **a**, **b** HeLa cells stably expressing TOMM20-mCherry(red) and mito-GFP (green) were treated with normoxia (21% O_2_) or hypoxia (1% O_2_) for 12 h, 24 h. Mitochondrial morphology was visualized by confocal microscopy (**a**). Right images show enlargements of the boxed areas in the left images. Mitochondrial morphology was quantified (**b**) according to the criteria detailed in “Methods”. *n* = 3 independent experiments. Data are presented as mean ± SEM, statistical significance was assessed by a two-way ANOVA. **c**–**e** HeLa cells were exposed to normoxia or hypoxia for 12 h or 24 h, then were by high pressure freezing and analyzed by transmission electron microscopy (TEM). Representative TEM images were shown, the red arrowhead: mitochondrion (**c**). The relative mitochondrial size (*n* = 40 mitochondria) was analyzed by ImageJ software (**d**). Mitochondrial cristae per mitochondrial area were quantified (**e**). “N”: normoxia, “H”: hypoxia. Data with error bars are presented as mean ± SD, statistical significance was assessed by a one-way ANOVA. **f**, **g** HeLa cells co-expressing mito-GFP (green) and TOMM20-mCherry (red) were treated with normoxia or hypoxia for 24 h, and the events of mitochondrial fusion were displayed (**f**). White arrowhead indicates mitochondrion-mitochondrion fusion site. The duration of mitochondrial fusion events in time-lapse confocal images was then counted (**g**). Error bars indicate the mean ± SD of the experiments, *n* = 21 mitochondrial fusion events, statistical significance was assessed by two-tailed *t*-test. **h**–**j** HeLa cells expressing mito-DsRed or photoactivable GFP (mito-PA-GFP) were treated with normoxia or hypoxia for 24 h, representative images were shown (**h**). The number of mitochondrial fission and fusion events within 20 min was then counted (**i**), and the ratio of mitochondrial fusion events to fission events was further calculated (**j**). 20 mitochondria were counted in each experiment. *n* = 3 independent experiments. Data are presented as mean ± SEM, statistical significance was assessed by a two-way ANOVA. *P* values are indicated in the figure. Source data are provided as a  Source Data file.

In response to hypoxia, the mitochondrial respiration and dynamics are impaired^31^. To further investigate the effect of hypoxia on mitochondrial morphology, HeLa cells co-expressing TOMM20-mCherry (mitochondrial outer membrane marker) and mito-GFP (mitochondrial matrix marker) were analyzed by confocal microscopy with Airyscan. Under normoxia, most mitochondria were tubular (Fig. 1a and b). In contrast, under hypoxia, some mitochondria were fragmented and most mitochondria became large spherical mitochondria that are displayed as a large circle rather than donut-shaped mitochondria as observed by TOMM20-mCherry fluorescence (Fig. 1a and b). These data suggest that the formation of large spherical mitochondria is not the cluster of many small mitochondria, consistent with the previous reports^31,32^. In addition, transmission electron microscope (TEM) analysis revealed that hypoxia led to the formation of large spherical mitochondria, which contain the outer mitochondrial membrane, inner mitochondrial membrane and mitochondrial matrix, and have fewer mitochondrial cristae compared with normal mitochondria (Fig. 1c–e), suggesting that large spherical mitochondria are not mitochondria-derived structures such as MDVs^33^, MDCs^34^, or SPOTs^35^. Therefore, we refer to “large spherical mitochondrion” as “megamitochondrion”. It is a large sphere (usually >2 μm in width and length) containing the outer mitochondrial membrane, inner mitochondrial membrane and mitochondrial matrix, and its ultrastructure is remolded (mitochondrial cristae are reduced and abnormal).

We then characterized the process of megamitochondria formation in live HeLa cells by time-lapse confocal imaging. Under normoxia, mitochondrial fusion process including contacts, fusion and remolding of two tubular mitochondria took ~12 s, whereas under hypoxia, the fusion process of two large mitochondria took longer than 30 s (Fig. 1f and g, Supplementary movie 1 and Supplementary movie 2), indicating that mitochondrial fusion process is slower under hypoxia than under normoxia. Moreover, inter-mitochondrial contacts were followed by subsequent fusion of mitochondria (Figs. 1f, g and S1a), which is also confirmed by HIS-SIM (High Sensitivity Structured Illumination Microscopy) imaging in live HeLa cells (Fig. S1a, Supplementary movie 3). In addition, Western blotting analysis showed that under hypoxia (6 h or 12 h), the mitochondrial fission factors DRP1, phosphorylated DRP1 (p-DRP1), FIS1 and the mitochondrial fusion factor OPA1 were markedly decreased, and the mitochondrial fusion factors MFN2 were slightly decreased (Fig. S1b and S1c), suggesting that both mitochondrial fission and fusion may be decreased under hypoxia. Furthermore, photoactivable GFP assay revealed that both mitochondrial fusion and fission rates are decreased but the ratio of mitochondrial fusion to mitochondrial fission were increased under hypoxia (Fig. 1h–j). In addition, confocal imaging showed *MFN1/2* DKD (*MFN1* and *MFN2* double knockdown) or *OPA1* knockdown cells displayed fully fragmented mitochondria under both normoxic and hypoxic conditions (Fig. S1d–g), suggesting that mitochondrial fusion is essential for megamitochondrial formation. The opening of mitochondrial membrane permeability transition (MPT) pores could lead to mitochondrial swelling^36^. Confocal imaging showed that cyclosporin A (inhibitor of MPT pore opening) treatment did not inhibit megamitochondria formation under hypoxia (Fig. S1h and S1i). These data suggest that megamitochondria formation under hypoxia is due to the increased ratio of mitochondrial fusion to mitochondrial fission, but not due to mitochondrial swelling or clustering. Interestingly, Zhou et al. also reported megamitochondria induced by cold exposure in *Sel1L*^−/−^ brown adipocytes are mostly due to the increased mitochondrial fusion^37^. In addition, confocal imaging showed that a small number (~8%) of megamitochondria recovered to normal/tubular mitochondria within 2 h of reoxygenation after 24 h of hypoxia (Fig. S1j), whereas the vast majority of megamitochondria remained unchanged.

Overall, hypoxia reprograms tubular mitochondria into megamitochondria by increasing the ratio of mitochondrial fusion to fission.

### Hypoxia promotes mitochondria-lysosome contacts and megamitochondria engulfing lysosome (MMEL)

**Fig. 2:**
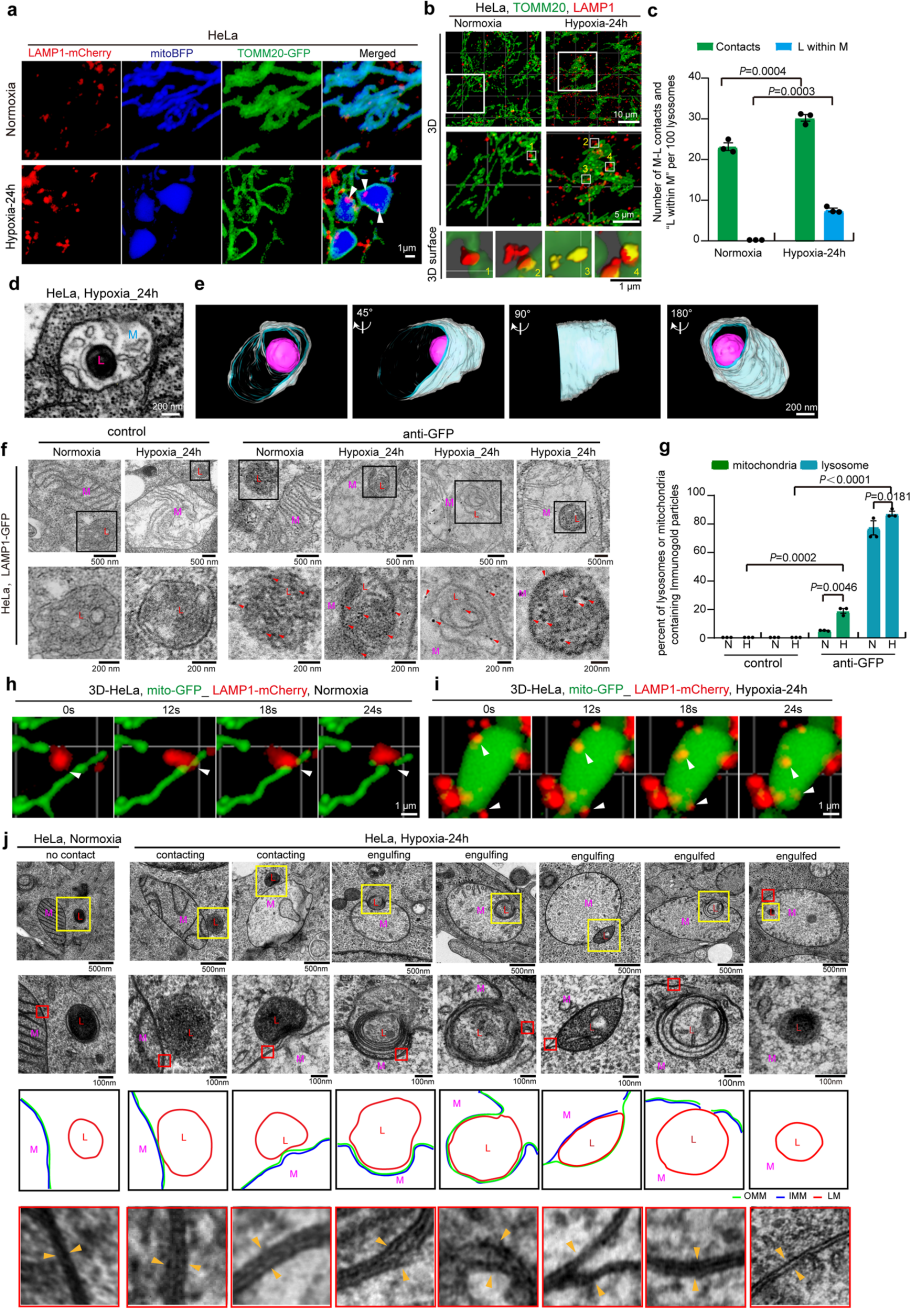
Hypoxia promotes mitochondria-lysosome contacts and induces lysosome entering into megamitochondria. **a** HeLa cells expressing LAMP1–mCherry, TOMM20-GFP and mito-BFP were treated with normoxia or hypoxia for 24 h, and analyzed by confocal microscopy. Representative images were shown, white arrowhead: lysosome. **b**, **c** HeLa cells were treated with normoxia or hypoxia for 24 h, and then immunostained with anti-LAMP1 and anti-TOMM20 antibodies, and analyzed by 3D imaging with confocal microscopy with Airyscan (**b**). The bottom images are mitochondria (green) and lysosome (red) which were displayed using 3D surface reconstructions of the middle images. The events of mitochondria-lysosome contacts (M-L contacts) and lysosome within megamitochondria (L within M) from 10 cells were then quantified (**c**) according to the criteria detailed in “Methods”, and the number of “M-L contacts” and “L within M” per 100 lysosomes was displayed. **d**, **e** HeLa cells treated with hypoxia (24 h) were analyzed by Focused ion beam/scanning electron microscopy (FIB-SEM), and representative images showing lysosome within mitochondria were displayed (**d**). 3D reconstruction and segmentation FIB-SEM images was shown (**e**). “M”: mitochondrion, “L”: lysosome. White, mitochondrial outer membrane; Cyan, mitochondrial inner boundary membrane; Purple Red, lysosome. **f**, **g** HeLa cells expressing LAMP1-GFP cells were treated with normoxia or hypoxia for 24 h, and then were fixed for GFP immunogold staining and immunoelectron microscopy analysis. red arrowhead: immuno-gold particle (**f**). The number of mitochondria and lysosomes containing immunogold particles was quantified, and 100 mitochondria or 100 lysosomes were analyzed for each experiment (**g**). **h**, **i** HeLa cells expressing LAMP1–mCherry (red) and mito-GFP (green) were treated with normoxia (**h**) or hypoxia (**i**) for 24 h, and then were tracked by time-lapse imaging with a confocal microscopy. Representative time-lapse confocal 3D images in living HeLa cells were displayed (*n* = 30 examples from 21 cells). **j** HeLa cells were exposed to normoxia or hypoxia for 24 h, then were fixed by high pressure freezing and analyzed by TEM. The red “L”: lysosome, the purple “M”: mitochondrion. All bars represent mean ± SEM, *n* = 3 independent experiments, statistical significance was assessed by a two-way ANOVA. *P* values are indicated in the figure. Source data are provided as a Source Data file.

Contacts between lysosomes and other organelles play an important role in mediating a variety of biological functions, including mitochondrial fission^23^. We then examined whether hypoxia affects mitochondria-lysosome contacts. Unexpectedly, confocal and three-dimensional (3D) imaging showed that hypoxia led to a marked increase in mitochondria-lysosome contacts (Fig. 2a–c). Surprisingly, some lysosomes (marked by LAMP1) were located inside megamitochondria under hypoxia but not under normoxia (Fig. 2a–c, Supplementary movie 4 and Supplementary movie 5). In addition, immunostaining with the lysosomal protease cathepsin D (CTSD) antibody or cell staining with the lysosomal dye LysoTracker Red confirmed that organelles within mitochondria under hypoxia were lysosomes or lysosome-related organelles (Fig. S2a–d). Furthermore, confocal Z-stacks analysis showed that the lysosome was indeed located within the megamitochondria in hypoxic cells (Fig. S2e, Supplementary movie 6). Furthermore, focused ion beam/scanning electron microscopy (FIB-SEM), 3D tomographic reconstruction and immunoelectron microscopy analysis showed that some lysosomes were located entirely within the megamitochondrion under hypoxia (Fig. 2d–g, and Supplementary movie 7 and Supplementary movie 8). These results suggest that the lysosome can enter into the megamitochondria under hypoxia. It should be noted that both single membrane and some multi-layered structured lysosomes were also labeled by immune-gold (Fig. 2f and S2f), consistent with the previous report that lysosomes can be characterized by specific features such as single membrane, intralumenal membrane swirls and intralumenal electron dense material under electron microscopy^38,39^.

Next, we investigated the process of lysosome entry into megamitochondria under hypoxia. Time-lapse confocal imaging showed that mitochondria (mito-GFP) transiently contacted lysosomes (LAMP1-mCherry), and certain mitochondria-lysosome contacts led to mitochondrial fission under normoxia (Fig. 2h). However, some lysosomes were able to contact and enter into the megamitochondrion under hypoxia, importantly, the megamitochondrion did not undergo fission during this process (Figs. 2i and S2g). Overall, hypoxia promotes mitochondria-lysosome contacts and induces lysosome to enter into megamitochondria.

Then, we investigated how lysosomes enter megamitochondria under hypoxia. It has been reported that lysosomal proteins translocate into mitochondria through the outer mitochondrial membrane pores formed by Mieap-BNIP3L complexes^40–42^. We found that BNIP3L knockdown had no effect on lysosome-megamitochondria contacts and lysosome entry into megamitochondria under hypoxia (Fig. S2h–j), suggesting that the penetration of lysosomes into megamitochondria is not dependent on the mitochondrial outer membrane pores formed by Mieap-BNIP3L complex. Therefore, we hypothesized that lysosomes may be engulfed into the megamitochondrion under hypoxia. Confocal imaging showed that certain lysosomes (labeled by LAMP1-mCherry) closely contacted megamitochondrion (labeled by mito-BFP or TOMM20-GFP) and caused invagination of the outer mitochondrial membrane (labeled by TOMM20-GFP) at the site of contact under hypoxia (Fig. S2g and S2k), suggesting that the megamitochondrion engulfs lysosomes. Further FIB-SEM and 3D tomographic reconstructions confirmed that lysosomes can be engulfed by megamitochondria in hypoxia (Fig. S3a and S3b, Supplementary movie 9 and Supplementary movie 10). Moreover, Time-lapse confocal imaging showed that the lysosome entering into megamitochondrion in live HeLa cells under hypoxia (Fig. S2g). Therefore, lysosomes can be engulfed by megamitochondria under hypoxia; we termed these events “megamitochondria engulfing lysosome (MMEL)”. To explore the steps of MMEL, we analyzed TEM images showing the events of MMEL. We captured events where the megamitochondrion contacts and engulfs the lysosome or with an engulfed lysosome (Fig. 2j). Interestingly, during MMEL, the mitochondrial membranes (outer and inner membranes) fold inward (invaginate), forming a cavity that fills with a lysosome (Figs. 2j, S3c and S3d). Then, the lysosome, surrounded by mitochondrial membranes, is pinched off from mitochondrial membranes as the ends of the inwardly folded mitochondrial membranes fuse (Fig. 2j). It should be noted that MMEL event also indicates a route of lysosome internalization.

Therefore, the process of hypoxia-induced MMEL may involve mitochondria-lysosome contact, engulfment, mitochondria-lysosome fusion, and rupture of mitochondrial membranes.

### Mitochondrial fusion and lysosomal maturation (activation) are required for MMEL

Next, we investigated whether mitochondrial dynamics affected MMEL. Confocal 3D imaging showed that *MFN1/2* DKD or *OPA1* KD caused mitochondrial fragmentation and increased mitochondria-lysosome contacts compared with control under normoxia (Figs. S3e and S3f). However, *MFN1/2* DKD or *OPA1* KD resulted in a reduction in MMEL under hypoxia (Fig. S3e and S3f). These results indicate that the fragmented mitochondria with small size can hardly induce MMEL events. In contrast, *DRP1* depletion induced the formation of long tubular and large spherical mitochondria, resulting in an increase in the number of megamitochondria and a significant increase in mitochondria-lysosome contacts and MMEL under normoxia or hypoxia (Fig. S3e, S3f, and S3h). It should be noted that *MFN1/2* DKD, *OPA1* KD or *DRP1* KD increased the number of lysosomes per cell (Fig. S3g). Overall, our results suggest that mitochondrial fusion-mediated megamitochondria are required for MMEL, which could be explained by the size inability of fragmented or even normal mitochondria to engulf the intact lysosome.

**Fig. 3:**
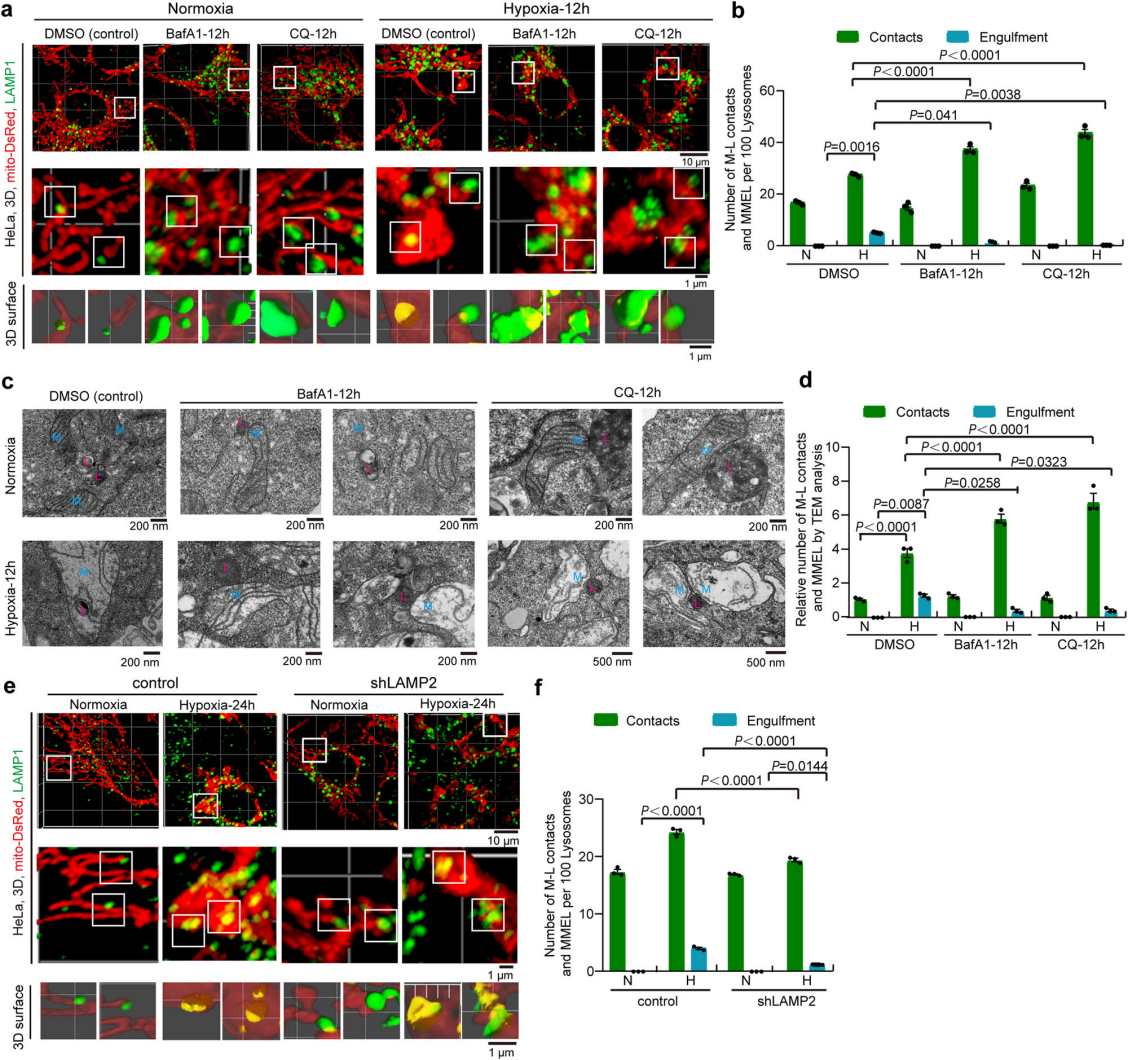
Mature lysosome is required for MMEL. **a**, **b** HeLa cells stably expressing mito-DsRed (mitochondria) were treated with normoxia or hypoxia in the presence of DMSO, Bafilomycin A1 (BafA1), or chloroquine (CQ) for 12 h. Cells were then immunostained with anti-LAMP1 (lysosome) antibody and were analyzed by 3D imaging with confocal microscopy with Airyscan. Mitochondria (red) and lysosome (green) were displayed using 3D surface reconstructions overlaid upon original data with Imaris software (**a**). The bottom images are 3D surface reconstructions of the middle images. Mitochondria-lysosome contacts (contacts) and MMEL (engulfment) from 10 cells were quantified in each experiment, and the number of M-L contacts and MMEL per 100 lysosomes was displayed (**b**). **c**, **d** HeLa cells were treated with normoxia or hypoxia in the presence of DMSO, BafA1 or CQ for 12 h. Cell samples were then analyzed by TEM. Representative TEM images containing mitochondria and lysosome were displayed (**c**). blue “M”: mitochondrion, red “L”: lysosome or lysosome-related organelle. Mitochondria-lysosome contacts (contacts) and MMEL (engulfment) per 100 mitochondria from 10 cells were then quantified in each experiment, and the relative data were displayed (**d**). **e**, **f** Control or *LAMP2* knockdown (sh*LAMP2*) HeLa cells stably expressing mito-DsRed (mitochondria) were treated with normoxia or hypoxia for 24 h. Cells were then fixed, immunostained with anti-LAMP1 (lysosome) antibody, and analyzed by 3D imaging with confocal microscopy with Airyscan. Mitochondria (red) and lysosome (green) were displayed using 3D surface reconstructions overlaid upon original data with Imaris software (**e**). The bottom images are 3D surface reconstructions of the middle images. Mitochondria-lysosome contacts (contacts) and MMEL (engulfment) from 10 cells were quantified in each experiment, and the number of M-L contacts and MMEL per 100 lysosomes was displayed (**f**). All bars represent mean ± SEM, *n* = 3 independent experiments, statistical significance was assessed by a two-way ANOVA. *P* values are indicated in the figure. Source data are provided as a Source Data file.

We also explored the effect of lysosome on MMEL under hypoxia. The lysosome is a highly acidified compartment generated and maintained by vacuolar ATPase (V-ATPase) and formed by a gradual maturation process^43^. Confocal imaging and Western blotting showed that lysosomes increased dramatically in HeLa cells exposed to hypoxia (Fig. S4a and S4b). Because Bafilomycin A (BafA1, a specific inhibitor of V-ATPase) and chloroquine (CQ, a lysosomotropic agent) can inhibit acidification of lysosomes and impair autophagosome-lysosome fusion, and thus block autophagy^44^, we then investigated whether BafA1 or CQ affects hypoxia-induced MMEL. Compared with control, treatment with BafA1 or CQ resulted in a remarkable increase in GFP-LC3 puncta, suggesting that BafA1 or CQ is functional to inhibit autophagy (Fig. S4c). Moreover, 3D confocal imaging and TEM analysis revealed that BafA1 or CQ treatment increased mitochondria-lysosome contacts but decreased MMEL under hypoxia (Fig. 3a–d). Also, BafA1 or CQ treatment did not alter mitochondrial size under normoxia and did not affect megamitochondria formation under hypoxia (Figs. 3a, 3c, S4d, and S4e), suggesting that acidification of lysosomes is essential for MMEL. In addition, LAMP1 and LAMP2 are important lysosomal membrane glycoproteins that are required for lysosomal membrane integrity^45^. We found that *LAMP1* or *LAMP2* KD dramatically reduced hypoxia-induced MMEL but had no effect on megamitochondria formation in HeLa cells (Figs. 3e, 3f, and S4f–j), indicating that lysosomal integrity is required for MMEL. These data suggest that lysosomal maturation (including acidification and integrity of lysosome) is essential for MMEL.

Overall, hypoxia-induced MMEL requires mitochondrial fusion and lysosomal maturation.

### Syntaxin17-SNAP29-VAMP7 complex contributes to the megamitochondria-lysosome contacts and MMEL under hypoxia

**Fig. 4:**
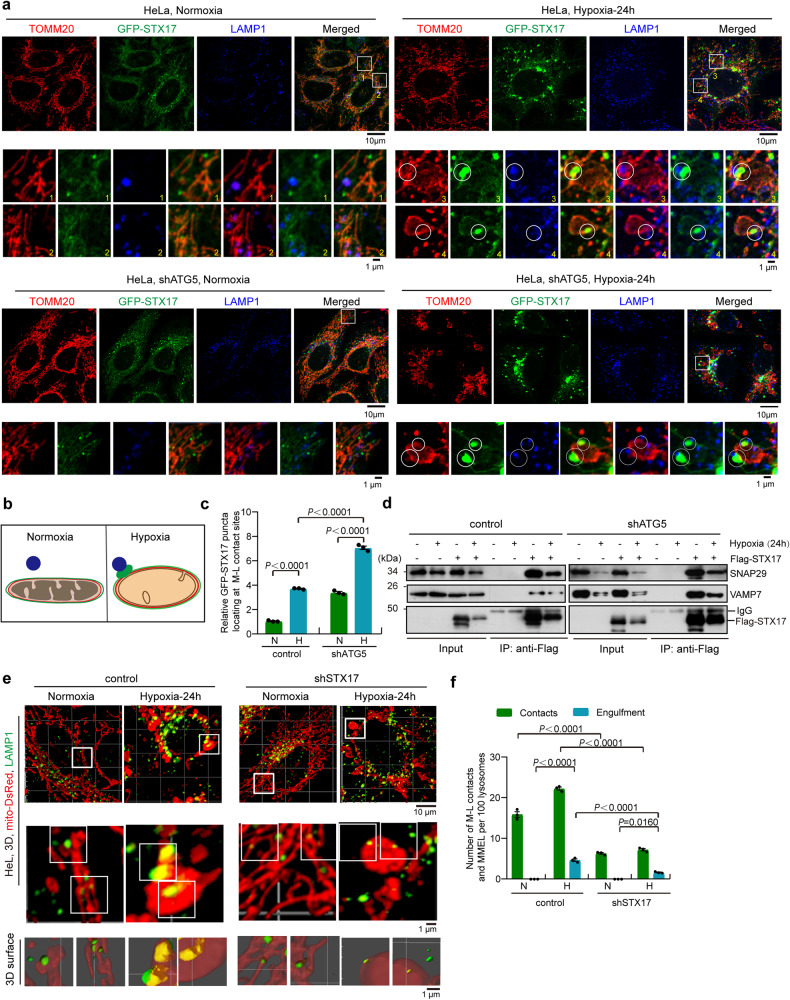
The role of STX17-SNAP29-VAMP7 complex in the mitochondria-lysosome contacts and MMEL. **a**–**c** WT or *ATG5* knockdown (sh*ATG5*) HeLa cells stably expressing GFP-STX17 (green) were exposed to normoxia or hypoxia for 24 h, and were immunostained with antibodies against TOMM20 (mitochondria, red), or LAMP1 (lysosome, blue), and were analyzed by confocal microscopy with Airyscan (**a**). The mode of localization of GFP-STX17 in “A” was drawn (**b**), green: GFP-STX17, red: TOMM20, blue: LAMP1 (lysosome). The number of GFP-STX17 puncta locating at megamitochondria-lysosome contact sites per cell were quantified (**c**), *n* = 10 cells for each experiment. **d** Control or *ATG5* knockdown HeLa cells were transfected with or without Flag-STX17, cell lysates were immunoprecipitated (IP) with anti-Flag M2 affinity gel, followed by Western blotting using anti-SNAP29, anti-VAMP7, or anti-Flag antibodies. **e**,**f** Control, *Syntaxin-17* knockdown (sh*STX17*) HeLa cells stably expressing mito-DsRed (mitochondria) were exposed to normoxia or hypoxia for 24 h, and were immunostained with an antibody against LAMP1 (lysosome), and were analyzed and imaged with confocal microscopy with Airyscan. Mitochondria (red) and lysosome (green) were displayed using 3D surface reconstructions overlaid upon original data with Imaris software (**e**). The bottom images are 3D surface reconstructions of the middle images (**e**). Mitochondria-lysosome contacts (contacts) and MMEL (engulfment) in control, *STX17* knockdown (**f**) (10 cells in each experiment) were quantified, and the number of M-L contacts and MMEL per 100 lysosomes was displayed. All bars represent mean ± SEM, *n* = 3 independent experiments, statistical significance was assessed by a two-way ANOVA. *P* values are indicated in the figure. Source data are provided as a  Source Data file.

Syntaxin 17 (STX17) cooperates with SNAP29 and VAMP7 (or VAMP8) to form STX17-SNAP29-VAMP7 (or VAMP8) complex and mediates autophagosome-lysosome fusion^46^. We therefore investigated the role of the STX17-SNAP29-VAMP7 complex in MMEL. Confocal imaging showed that under normoxia, a large fraction of GFP-STX17 (stably expressing) or endogenous STX17 (immunostaining) co-localizes with TOMM20 (mitochondrial marker) and little GFP-STX17 or endogenous STX17 co-localizes or contacts LAMP1 (lysosome marker) (Figs. 4a, 4b and S5a), suggesting that most STX17 localizes to mitochondria. Strikingly, under hypoxia, some STX17 accumulated and formed many puncta; moreover, some puncta were located at the mitochondria-lysosome contact sites (Figs. 4a–c and S5a). Because the STX17-SNAP29-VAMP7 complex primarily mediates autophagosome-lysosome binding and fusion^46^, we examined the localization of STX17 in *ATG5* knockdown cells (Fig. S5b), in which autophagosome formation is blocked, to investigated whether that the formation of STX17 puncta and STX17-SNAP29-VAMP7 complex are dependent on autophagosome-lysosome fusion. STX17 puncta were still located at the megamitochondria-lysosomes contact sites in *ATG5* knockdown cells under hypoxia (Figs. 4a–c). Furthermore, the co-IP assay showed that STX17 interacted with SNAP29 and VAMP7 in control or *ATG5* knockdown cells under hypoxia (Fig. 4d). These data suggest that the STX17-SNAP29-VAMP7 complex is involved in megamitochondria-lysosome contacts and MMEL under hypoxia. In addition, hypoxia caused a significantly decrease in VAMP7 and SNAP29 in *ATG5* knockdown cells compared with normoxia (Fig. S5c and S5d), probably because the STX17-SNAP29-VAMP7 complex might be degraded after hypoxia-induced MMEL.

We then examined the effect of the STX17-SNAP29-VAMP7 complex on MMEL. *STX17* knockdown (Fig. S5e) dramatically decreased mitochondria-lysosome contacts and MMEL (Fig. 4e and f); in contrast, overexpression of GFP-STX17 significantly increased mitochondria-lysosome contacts and MMEL (Fig. S5f and S5g). These results suggest that STX17 regulates the megamitochondria-lysosome contacts and hypoxia-induced MMEL. Furthermore, overexpression of GFP-STX17 significantly increased mitochondria-lysosome contacts in both *MFN1/2* DKD and WT (control) cells but failed to rescue hypoxia-induced MMEL in *MFN1/2* DKD cells (Fig. S5f and S5g). In addition, *SNAP29* or *VAMP7* knockdown significantly inhibited mitochondria-lysosome contacts and MMEL under hypoxia (Fig. S6a–d). In addition, knockdown of *STX17*, *SNAP29* or *VAMP7* had no effect on megamitochondria formation under hypoxia (Fig. S6e). These data suggest that the STX17-SNAP29-VAMP7 complex regulates megamitochondria-lysosome contacts and MMEL under hypoxia. It should be noted that some megamitochondria in *STX17* knockdown cells under hypoxia may also undergo mitochondrial fusion and fission processes that remodel megamitochondria into a different morphology, but the majority of megamitochondria remain unchanged (Fig. S6f).

In addition, TEM imaging showed close contact and fusion between lysosomal and mitochondrial membranes during MMEL [Fig. 2j (the sixth panel), and S7a], suggesting that mitochondria-lysosome membrane fusion may be associated with MMEL. We then used the bimolecular fluorescence complementation (BiFC) assay (directly visualize protein-protein interaction in cells) to determine the role of the STX17-SNAP29-VAMP7 complex in mitochondria-lysosome contacts or fusion. In control or *ATG5* knockdown cells in which STX17-VN and SNAP29-VC were co-expressed, little fluorescence was detected under normoxia, but remarkable fluorescence was seen under hypoxia (Fig. S7b), suggesting that STX17-SNAP29 interaction is dramatically increased under hypoxia, suggesting that the STX17-SNAP29-VAMP7 complex is increased under hypoxia, which could promote megamitochondria-lysosome membrane contacts and fusion. Moreover, STX17-ΔNTD, which lacks the N-terminal transmembrane domain and is a dominant-negative mutant of STX17^47^, could still be localized to the megamitochondria-lysosome contact sites and significantly inhibited MMEL, however, without affecting megamitochondria-lysosome contacts under hypoxia (Fig. S7c–e), suggesting that STX17 is involved in megamitochondria-lysosome membrane fusion. Thus, megamitochondria-lysosome membrane fusion may be involved in the process of MMEL under hypoxia.

Overall, the STX17-SNAP29-VAMP7 complex accumulates at megamitochondria-lysosome contact sites and contributes to mitochondria-lysosome membrane contacts and MMEL under hypoxia.

### Lysosome membrane is ruptured to release lysosomal proteases into megamitochondria after MMEL

**Fig. 5:**
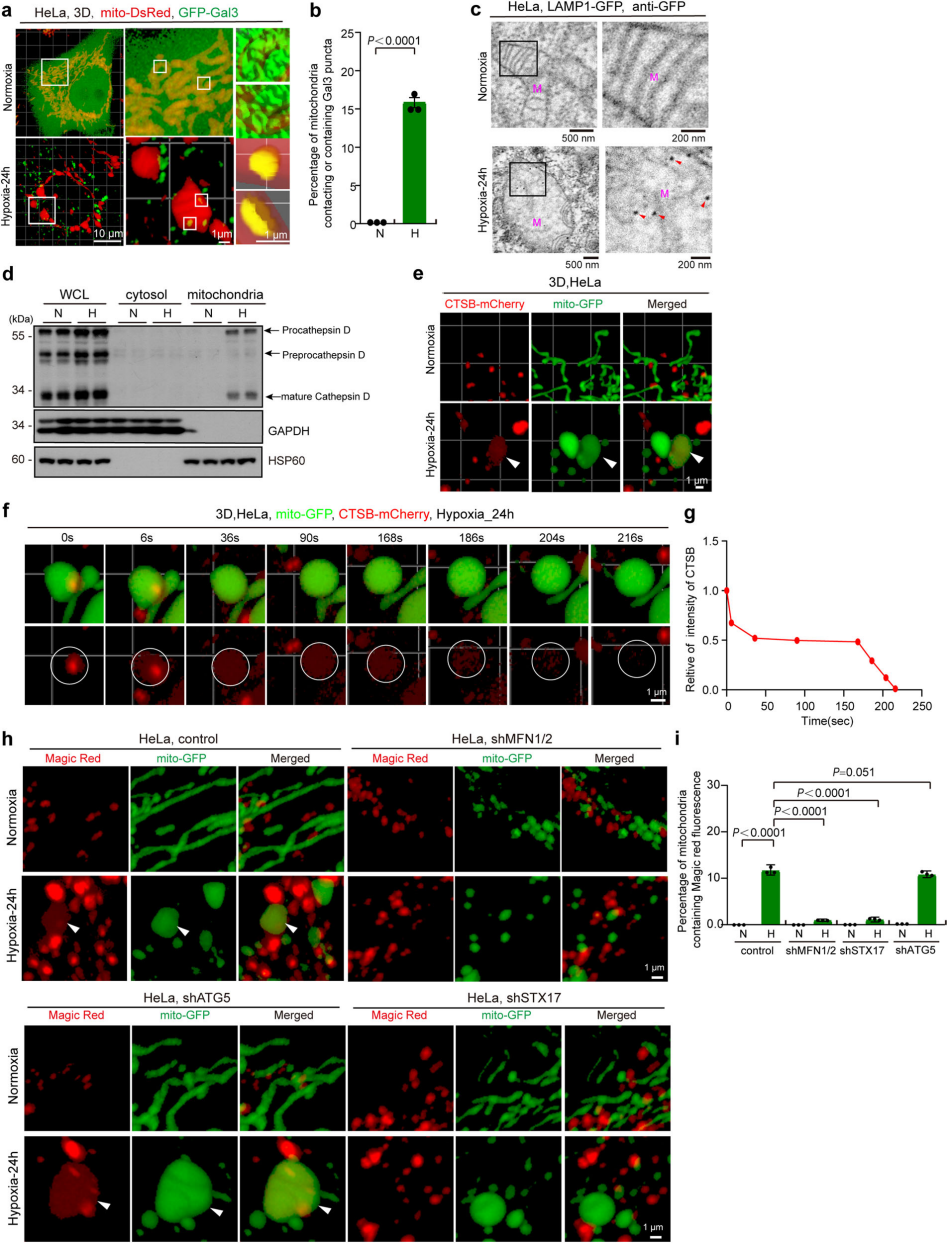
Lysosomal membrane is ruptured and lysosomal proteases are released within megamitochondria after MMEL. **a**, **b** HeLa cells co-expressing mito-DsRed (mitochondria) and GFP-Gal3 treated with normoxia or hypoxia for 24 h. Cells were then fixed and analyzed by 3D imaging with confocal microscopy with Airyscan, and the images of 3D surface reconstructions were shown (**a**). Mitochondria (mito-DsRed) contacting and containing GFP-Gal3 puncta from 10 cells were quantified in each experiment, and the percentage was displayed (**b**). Error bars indicate the mean ± SD of the experiments, *n* = 3 independent experiments, statistical significance was assessed by two-tailed *t*-test. **c** HeLa cells expressing LAMP1-GFP cells were treated with normoxia or hypoxia for 24 h, and then were fixed for GFP immuno-gold staining and immunoelectron microscopy analysis. Red arrowhead: immuno-gold particle. **d** HeLa cells were exposed to normoxia or hypoxia for 24 h, and then were extracted for the subcellular fractionation assay. The cytosolic and mitochondrial fractions were then analyzed by Western blotting using anti-cathepsin D, anti-GAPDH, or anti-HSP60 antibodies. Whole cell lysates (WCL) were used as control. **e** HeLa cells expressing CTSB–mCherry (red) and mito-GFP (green) were treated with normoxia or hypoxia for 24 h, 3D images of mitochondria-lysosome contacts or lysosome within megamitochondria in living HeLa cells were displayed. **f**, **g** Living HeLa cells expressing CTSB–mCherry (red) and mito-GFP (green) were treated with hypoxia for 24 h, and then were tracked and imaged with confocal microscopy with Airyscan. 3D images in living cells were displayed (**f**). The fluorescence intensity of lysosomal enzyme within megamitochondria in “I” was analyzed by image J software (**g**). **h**, **i** Control, *MFN1/2*, *ATG5* or *STX17* knockdown (sh*MFN1/2*, sh*STX17* or sh*ATG5*) HeLa cells stably expressing mito-GFP (green) were treated with normoxia or hypoxia for 24 h, then incubated with Magic Red for 1 h, and analyzed and imaged with confocal microscopy with Airyscan (**h**). The number of mitochondria containing Magic Red fluorescence per 1000 mitochondria from 10 cells were then quantified, and the percentage was displayed (**i**). *n* = 3 independent experiments. Data are presented as mean ± SEM, statistical significance was assessed by a two-way ANOVA. *P* values are indicated in the figure. Source data are provided as a Source Data file.

Next, we investigated whether the membrane integrity of lysosomes within mitochondria is impaired after MMEL under hypoxia. We used galectins-3, a marker of lysosomal membrane permeabilization, to detect the integrity of the lysosomal membrane^48^. The number of GFP-galectins-3 puncta in contact with or engulfed by megamitochondria was significantly increased under hypoxia (Fig. 5a and b), suggesting that the membrane integrity of some lysosomes is impaired during/after MMEL under hypoxia. Furthermore, GFP-LAMP1 expressed HeLa cells were analyzed by immunoelectron microscopy. Immuno-gold particles (indicative of GFP-LAMP1) could be observed in megamitochondria without intact lysosomal structure under hypoxia but not under normoxia (Fig. 5c), suggesting that the lysosomal membrane may be ruptured after MMEL under hypoxia. Then, we investigated whether lysosomal proteases release into megamitochondria after MMEL. Subcellular fractionation assays and subsequent Western blotting analysis showed that hypoxia increased the levels of cathepsin D (CTSD, a major lysosomal protease) in the mitochondrial fraction (Fig. 5d and e). In addition, some cathepsin B (CTSB, another major lysosomal protease) could locate and diffuse in mitochondria (Fig. 5e). Moreover, time-lapse confocal imaging showed that lysosomes labeled with CTSB-mCherry were able to contact and enter megamitochondria, followed by the release and diffusion of CTSB-mCherry within megamitochondria (Fig. 5f, Supplementary movie 11). It should be noted that the released lysosomal protease CTSB does not remain long in mitochondria and can be degraded in mitochondria (Fig. 5f and g). In addition, GFP-LAMP1 (lysosomal membrane protein) is also reduced in hypoxic cells (Fig. S8a and S8b). These data indicate that lysosomal proteases could release into megamitochondria after MMEL under hypoxia.

Then, we investigated whether released lysosomal proteases are active in mitochondria. We used the Magic Red assay (the Magic Red substrate in this assay fluoresces red upon cleavage by active cathepsin enzymes) to monitor cathepsin B activity in cultured cells expressing mito-GFP. Under normoxia, red fluorescence (active cathepsin B indicator) was not colocalized with green fluorescence (mitochondrial indicator) in control, sh*MFN1/2*, sh*ATG5*, or sh*STX17*-HeLa cells (Fig. 5h and i), suggesting that active cathepsins locate mainly in lysosomes but not mitochondria. In contrast, under hypoxia, ~12% (most of which are megamitochondria) of mitochondria in control HeLa cells showed diffused Magic Red fluorescence (Fig. 5h and i), suggesting that lysosomal proteases are active in mitochondria after MMEL and also indicating that hypoxia induces MMEL in the cell. However, *MFN1/2* DKD or *STX17* KD but not *ATG5* KD dramatically reduced the number of mitochondria that contained Magic Red fluorescence under hypoxia (Fig. 5h and i), suggesting that the mitochondria containing Magic Red fluorescence are due to MMEL but not mitophagy. It should be noted that the activity of lysosomal proteases in mitochondria is probably not high or does not last long. Thus, these data suggest that the engulfed lysosome is active (at least partially) in mitochondria to digest mitochondrial proteins.

Therefore, lysosome membrane integrity is impaired to release lysosomal proteases into megamitochondria after MMEL.

### MMEL cooperates with mitochondrial proteases to mediate mitochondrial self-digestion (MSD) under hypoxia

**Fig. 6:**
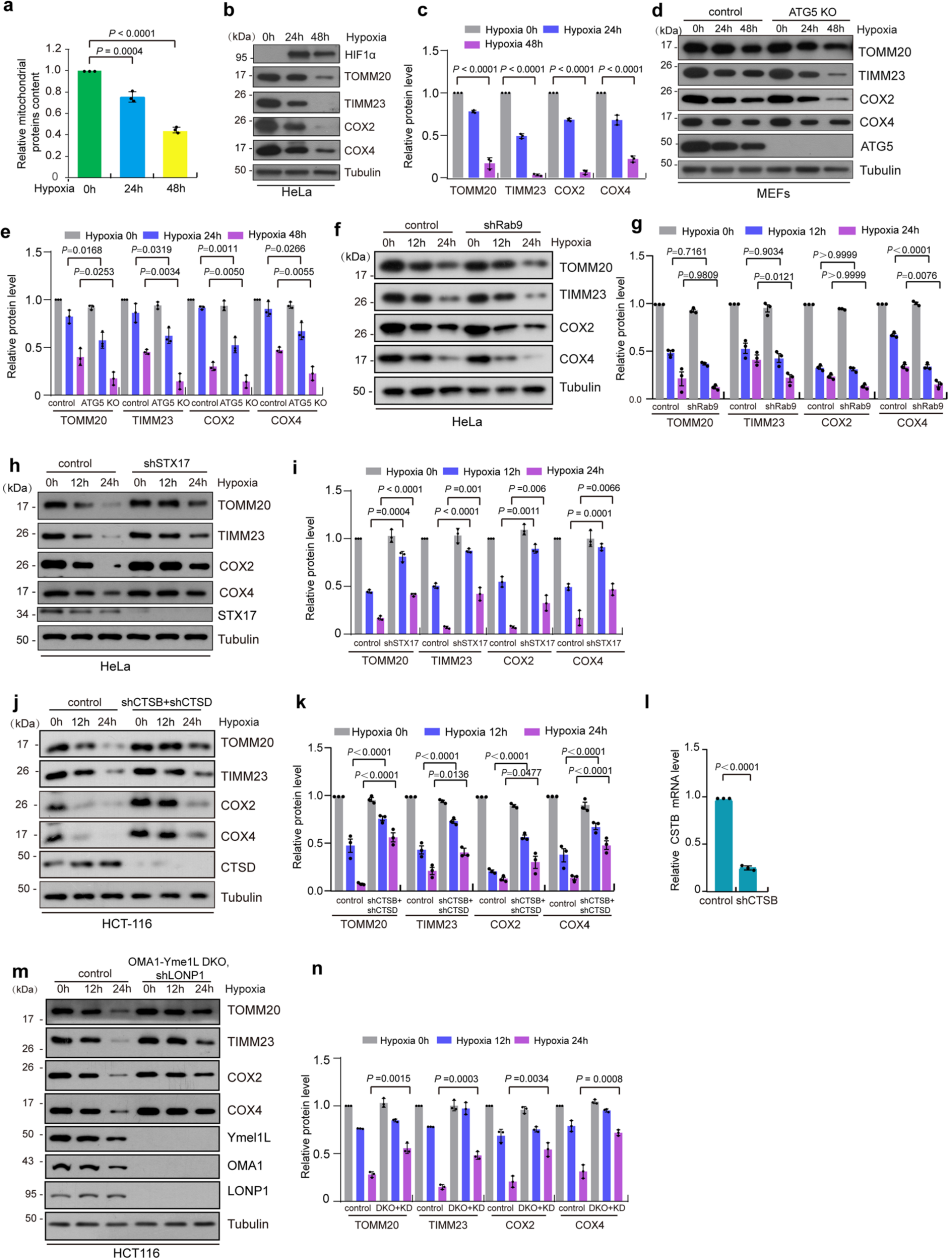
MMEL and mitochondrial proteases contribute to the degradation of mitochondrial proteins. **a** HeLa cells were treated with hypoxia for 0 h (normoxia), 24 h or 48 h, and then collected and used for mitochondrial purification. The concentration of mitochondrial proteins was measured by the Bradford assay. **b**, **c** HeLa cells were treated with hypoxia for 0 h (normoxia), 24 h, and 48 h. The whole cell lysates were then analyzed by Western blotting with the indicated antibodies (**b**). The relative protein levels were further evaluated by densitometry analysis using ImageJ software (**c**). **d**, **e** WT or *ATG5* knockout (KO) MEFs were treated with hypoxia for 0 h (normoxia), 24 h, and 48 h. Cell lysates were then analyzed by Western blotting with the indicated antibodies (**d**). The relative protein levels were further evaluated by densitometry analysis using ImageJ software (**e**). **f–i** Control or *Rab9* knockdown (sh*Rab9*) HeLa cells were exposed to hypoxia for 0 h (normoxia), 12 h, or 24 h. Control or *STX17* knockdown (sh*STX17*) HeLa cells were exposed to hypoxia for 0 h (normoxia), 12 h, or 24 h. Cell lysates were then analyzed by Western blotting with the indicated antibodies (**f**, **h**). The relative protein levels were further evaluated by densitometry analysis using ImageJ software (**g**, **i**). **j–n** Control, *cathepsin D* and *cathepsin B* double knockdown (sh*CTSB*+sh*CTSD*) HCT116 cells were exposed to hypoxia for 0 h (normoxia), 12 h, and 24 h. Control (empty vector), *OMA1-Yme1L* double knockout (DKO) plus *LONP1* knockdown (sh*LONP1*) HCT116 cells were exposed to hypoxia for 0 h (normoxia), 12 h, and 24 h. Cell lysates were then analyzed by Western blotting using the indicated antibodies (**j**, **m**). The relative protein levels were further evaluated by densitometry analysis using ImageJ software (**k**, **n**). The relative levels of *CTSB* mRNA were analyzed by quantitative RT-PCR (**l**). Bars of (**a**, **c** and **l**) represent mean ± SD, *n* = 3 independent experiments, statistical significance was assessed by a one-way ANOVA. Bars of (**e**, **g**, **i**, **k** and **n**) represent mean ± SD, *n* = 3 independent experiments, statistical significance was assessed by two-tailed *t*-test. *P* values are indicated in the figure. Source data are provided as a Source Data file.

Since lysosomal proteases could release into mitochondria and be active after MMEL, we investigated the role of MMEL on mitochondrial protein degradation. We separated and purified mitochondrial fractions from normoxic and hypoxic HeLa cells, and found that the amount of total mitochondrial proteins was reduced in hypoxic to ~75% (24 h) and 43% (48 h) of those in normoxic cells (Fig. 6a). We also performed Western blotting analysis of purified mitochondria with antibodies against TOMM20 (mitochondrial outer membrane protein), TIMM23 (mitochondrial inner membrane space protein), COX2 (mitochondrial inner membrane protein encoded by mitochondrial DNA), and COX4 (mitochondrial inner membrane protein encoded by nuclear DNA). Mitochondrial proteins were significantly decreased in HeLa, HCT116, or MCF7 cells under prolonged hypoxic conditions (24 h or 48 h) (Figs. 6b, c, and S8c–S8f), suggesting that hypoxia induces the degradation of mitochondrial proteins. We next investigated whether hypoxia-induced mitochondrial proteins degradation is dependent on traditional autophagosome-mediated mitophagy. *ATG5* KD markedly inhibits autophagosome-mediated mitophagy detected by mito-Keima assay under hypoxia (Fig. S8g and S8h). Also, mito-Keima assay and confocal imaging showed that about 29.47% mitochondria were undergoing mitophagy (indicated by red puncta) and about 49.61% of mitochondria were megamitochondria in a cell under hypoxia (Fig. S8i and S8j); but the ratio of the area of mitochondria undergoing mitophagy or megamitochondria to the area of the total mitochondria was 0.11 or 0.67 under hypoxia, respectively (Fig. S8i and S8j). In addition, *ATG5* KD or knockout (KO) did not inhibit mitochondrial protein degradation under hypoxia and, in fact, even slightly promoted it compared with control (Figs. 6d, 6e, S8k and S8l). Moreover, TEM analysis showed that the electron density of *ATG5* KD mitochondria under hypoxia was significantly lower than that of control mitochondria (Fig. S8m and S8n), further indicating that *ATG5* KD does not inhibit hypoxia-induced mitochondrial degradation. Moreover, *ATG5* KD did not affect MMEL under hypoxia (Fig. S9a and S9b). In addition, Dhingra et al. reported an alternative mitophagy pathway mediated by Ulk1/Rab9^49^. We found that *Rab9* KD had no effect on MMEL and did not inhibit mitochondrial protein degradation under hypoxia (Figs. 6f, g and S9c-S9e). These data suggest that other pathways (such as MMEL) besides autophagosome-dependent and Rab9-mediated mitophagy are responsible for mitochondrial degradation under hypoxia.

Next, we investigated the role of MMEL in mitochondrial degradation. We assessed the effect of STX17, which regulates MMEL (Fig. 4e and f), on mitochondrial degradation. *STX17* KD markedly inhibited the degradation of mitochondrial proteins including TOMM20, TIMM23, COX2, and COX4 under hypoxia (Fig. 6h and i), suggesting that MMEL contributes to mitochondrial degradation under hypoxia, and indicating that lysosome within megamitochondria is responsible for MMEL-mediated mitochondrial degradation. Then, we investigated whether lysosomes activity is required for hypoxia-induced mitochondrial degradation during MMEL. We used BafA1 to inhibit lysosome acidification and activity. Western blotting analysis showed that BafA1 treatment strongly inhibited hypoxia-induced degradation of mitochondrial proteins (Fig. S9f and S9g). In addition, TEM assays showed that hypoxia resulted in a reduction in mitochondrial cristae and mitochondrial mass (as reflected by the reduced electron density of hypoxic mitochondria) (Fig. S10a–c), but BafA1 treatment significantly prevented the reduction (Fig. S10a–c). In addition, HIS-SIM imaging showed that mitochondrial cristae (labeled by PKMDR, which stains the mitochondrial inner and cristae membranes) were reduced and remolded under hypoxia after lysosome (labeled by LAMP1-mCherry) engulfment (Fig. S10d). Furthermore, depletion of lysosomal proteases (cathepsin D plus cathepsin B) remarkably blocked the degradation of mitochondrial proteins under hypoxia (Fig. 6j–l). Therefore, hypoxia-induced MMEL contributes to mitochondrial degradation, in a process that depends on lysosomal incorporation and lysosomal proteases within the megamitochondrion.

The mitochondrion itself contains ~40 mitochondrial proteases, we therefore investigated whether mitochondrial proteases are also activated and involved in the degradation of mitochondria during hypoxia. qRT-PCR analysis showed that hypoxia significantly increased *LONP1* mRNA levels, but not altered mRNA levels of other mitochondrial proteases (*AFG3L2*, SPG*7*, *Yme1L*, *OMA1*, *PARL*, *HTRA2*, *CLPP*, *CLPX*, *IMMP1L* and *IMMP2L*) and other mitochondrial proteins (*MFN1*, *MFN2*, *HSPD1*, *COX4L1*, *COX2*, *TOMM20* and *TIMM23*) (Fig. S11a). Western blotting analysis showed that protein levels of mitochondrial protease LONP1, AFG3L2 and PARL, but not Yme1L, OMA1 and HTRA2, were increased in cells exposed to hypoxia for 6 or 12 h (Fig. S11b–k). Moreover, OPA1 is markedly cleaved under hypoxia (Fig. S11d and S11f), indicating that hypoxia activates the mitochondrial proteases OMA1 and Yme1L because *OPA1* is a known substrate of OMA1 and Yme1L^50–52^. Similarly, hypoxia caused degradation of HAX1 (a substrate of HTRA2) and cleavage of PGAM5 (a substrate of PARL) (Fig. S11g–k), indicating that the activities of PARL and HTRA2 are also increased under hypoxia. Importantly, depletion of the various mitochondrial proteases (LONP1, Yme1L, plus OMA1) remarkably blocked the degradation of mitochondrial proteins under hypoxia (Fig. 6m and n). Altogether, hypoxia activates a number of mitochondrial proteases, leading to the degradation of mitochondrial proteins. Also, the number of hypoxia-induced MMEL events was significantly reduced in *LONP1* knockdown cells (Fig. S12a–c), suggesting that hypoxia-activated mitochondrial proteases promote MMEL.

Overall, hypoxia induces not only traditional autophagosome-dependent mitophagy but also MMEL, lysosomal incorporation and activation within mitochondria, and activation of mitochondrial proteases. This sequence of events initiates a type of mitochondrial degradation that mediates MMEL and occurs within mitochondria but not within autophagosome (the mitochondria are engulfed), which is why we name it as “mitochondrial self-digestion (MSD)” or “mitochondrial self-autophagy (mitoselfphagy)”. We show that activation of MSD by hypoxia leads to digestion of mitochondrial proteins and other molecules within the mitochondria by activating engulfed lysosomal proteases and mitochondrial proteases. Thus, unlike traditional mitophagy, MSD (or mitoselfphagy) is independent of autophagosome formation and the classical mitophagy pathway.

Also, mass spectrometry analysis showed that most mitochondrial proteins located in different mitochondrial compartments were decreased to varying degrees after hypoxia (Fig. S12d–g). Some mitochondrial proteins, such as OPA1, COX2, MIC19, VDACs, TIMM13, GRPEL1, GRPEL2, NDUFS4, and APOO, were greatly decreased under hypoxic conditions (Fig. S12d–g). However, certain proteins, such as BNIP3 and BNIP3L, which are transcriptionally regulated by HIF-1α, were increased under hypoxia (Fig. S12d). In addition, some mitochondrial proteins such as TOMM70, TOMM22, APOE, MIC27, CHCHD2, POLG2, NDUFA2, COX5A, and COA3, remained almost unchanged after hypoxia (Fig. S12d–g). These data show that MSD degrades different mitochondrial proteins to different extents, while traditional mitophagy degrades total mitochondria.

Thus, MMEL cooperates with mitochondrial proteases to mediate MSD under hypoxia.

### MMEL-mediated MSD promotes mitochondrial ROS production

**Fig. 7:**
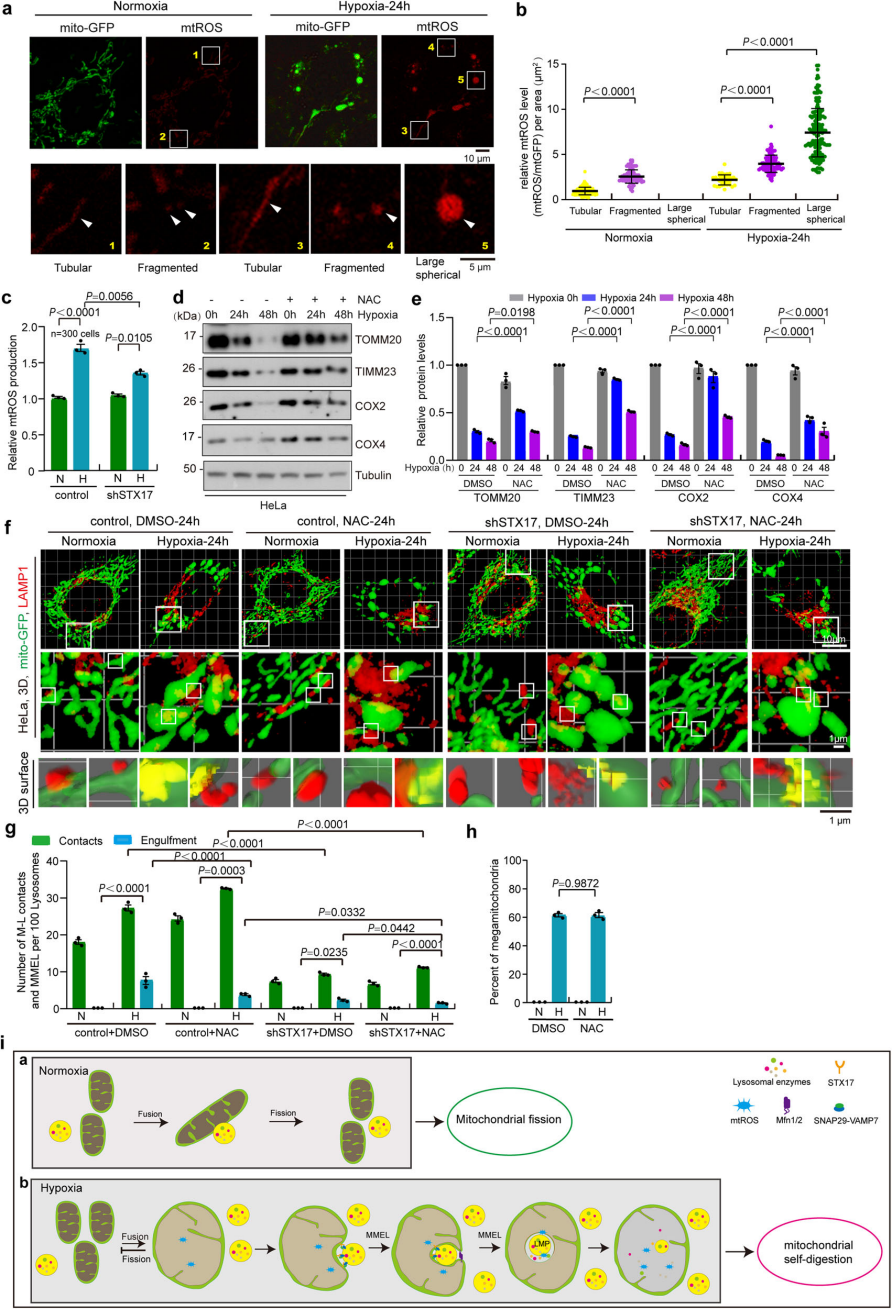
MMEL promotes mitochondrial ROS production under hypoxia. **a**–**c** Control or *STX17* knockdown (sh*STX17*) HeLa cells expressing mito-GFP were cultured in normoxia or hypoxia for 24 h and then stained with mitoSOX (**a**). The relative mitoSOX fluorescence intensity (ratio to mito-GFP fluorescence intensity) of the fragmented, tubular, or large spherical mitochondria (megamitochondria,) in cells described in (**a**) was further analyzed. 300 mitochondria from 30 cells under normoxia or hypoxia were quantified (**b**). **c** The relative level of the mitoSOX fluorescence intensity was displayed. **d**, **e** HeLa cells treating with DMSO or N-acetyl-L-cysteine (NAC) were cultured in or hypoxia for 0 h (normoxia), 24 h, or 48 h. Cell lysates were analyzed by Western blotting using the indicated antibodies (**d**). The relative protein levels were evaluated by densitometry analysis (**e**). Error bars indicate the mean ± SD of the experiments, *n* = 3 independent experiments, statistical significance was assessed by two-tailed *t*-test. **f**–**h** Control or *STX17* knockdown (sh*STX17*) HeLa cells stably expressing mitoGFP (mitochondria) treating with DMSO (control) or N-acetyl-L-cysteine (NAC) were cultured in or hypoxia for 0 h (normoxia), 24 h, and then immunostained with anti-LAMP1 antibodies, and analyzed by 3D imaging with confocal microscopy with Airyscan. Mitochondria (green) and lysosome (red) were displayed using 3D surface reconstructions (**f**). The events of mitochondria-lysosome contacts and megamitochondria engulfing lysosome (MMEL) from 10 cells were quantified in each experiment, and the number of M-L contacts and MMEL per 100 lysosomes was displayed (**g**). The number of megamitochondria described in “**f**” were also quantified (**h**) according to the criteria detailed in “Methods”. **i** Under normoxia (**a**), mitochondria-lysosome contacts contribute to mitochondrial fission. Under hypoxia (**b**), many normal mitochondria contact (mitochondria-mitochondria contact, M-M contact) and fuse to form megamitochondria, which then contact and engulf lysosomes with the help of the STX17-SNAP29-VAMP7 complex. Then lysosomes release some hydrolases to degrade mitochondrial proteins and other molecules. In addition, mitochondrial proteases (MP) are activated in hypoxia, which cooperates with MMEL to mediate the inverted mitophagy. Bars of (**b**, **c**, **g** and **h**) represent mean ± SEM, *n* = 3 independent experiments, statistical significance was assessed by a two-way ANOVA. *P* values are indicated in the figure. Source data are provided as a Source Data file.

Reactive oxygen species (ROS) are mainly produced in the mitochondria and play a number of roles in cells. Under hypoxia, mitochondria generate a large amount of ROS due to the imbalanced electron transport chain^27^. Therefore, we investigated the effects of MMEL-mediated MSD on mitochondrial ROS (mtROS) production. MitoSOX^TM^ Red staining assay showed that the mtROS level of megamitochondria (can MMEL) was significantly higher than that of fragmented (no MMEL) and tubular mitochondria (no MMEL) (Fig. 7a and b). Moreover, *STX17* knockdown, which decreases MMEL (Fig. 4e and f), resulted in a decrease in mtROS under hypoxia compared with control (Fig.7c). These data demonstrate that MMEL-mediated MSD promotes mtROS production under hypoxia. In addition, megamitochondria containing lysosomes showed increased mitoSOX (detection of mitoROS) or decreased TMRM (detection of mitochondrial membrane potential) fluorescence intensity (Fig. S13a–c), suggesting that mitochondria targeted for MMEL are more damaged. However, most cytochrome c remained in megamitochondria and just slightly amount of cytochrome c released (may be from tubular mitochondria) into the cytosol during MMEL under hypoxia (Fig. S13d and S13e), suggesting that mitochondrial damage induced by MMEL does not lead to cell death. In addition, the opening of mitochondrial membrane permeability transition (MPT) pores leads to the dissipation of the membrane potential, swelling and the release of cytochrome c^36^. We then investigated the relationship between MMEL and MPT pore. Confocal imaging showed that cyclosporin A (inhibitor of MPT pore opening) treatment did not block the MMEL under hypoxia (Fig. S13f and S13g), indicating that MMEL is not dependent on MPT pore opening.

Next, we investigated whether inhibition of ROS blocks MMEL-mediated MSD. The antioxidant N-acetyl-L-cysteine (NAC) was used to inhibit ROS, and NAC treatment significantly inhibited mitochondrial protein degradation under hypoxia (Fig. 7d and e), suggesting that inhibition of mtROS reduces MSD. In addition, NAC treatment significantly decrease MMEL but not megamitochondria-lysosome contacts and the number of megamitochondria under hypoxia (Fig. 7f–h), suggesting that increased mtROS promotes MMEL under hypoxia. In addition, NAC treatment markedly inhibited MMEL in control or *STX17* knockdown cells under hypoxia (Figs. 7f–h), suggesting that NAC treatment could inhibit MMEL formation in *STX17* knockdown cells. This is probably because *STX17* knockdown affects the megamitochondria-lysosome contacts, whereas NAC affects the ROS level of megamitochondria. It should be noted that hypoxia-reoxygenation also dramatically increases mtROS generation^53^, we will explore the effect of hypoxia-reoxygenation on MMEL and MSD in our future study.

Overall, MMEL-mediated MSD promotes mtROS production, which in turn promotes MMEL under hypoxic conditions.

## Discussion

Both mitochondria and lysosomes are critical for cellular homeostasis. Mitochondria and lysosomes are functionally interdependent, and the interactions between mitochondria and lysosomes play an important role in cellular metabolism and signal transduction. Here, we found that hypoxia induces megamitochondria formation, promotes mitochondria-lysosome contacts, and leads to megamitochondria engulfing lysosomes (MMEL) in mammalian cells. Moreover, hypoxia activates a number of mitochondrial proteases that cooperate with MMEL to initiate MSD (or mitoselfphagy) in an autophagosome-independent manner. MSD contributes to mitochondrial degradation and promotes mtROS production (Fig. 7i).

Mitochondrial fission facilitates mitophagy because small fragmented mitochondria are readily sequestered by autophagosomes and delivered to the lysosome for degradation^54^. In contrast, mitochondrial fusion promotes the formation of long tubular or megamitochondria that resist being engulfed by autophagosome, resulting in the inhibition of mitophagy. Interestingly, hypoxia induces the formation of both fragmented and megamitochondria (Fig. 1a–d), and the mito-Keima assay showed that most fragmented mitochondria undergo mitophagy, whereas megamitochondria resist to mitophagy under hypoxia (Fig. S8g). We also found that megamitochondria under hypoxia can engulf lysosomes themselves instead of being delivered to lysosomes for degradation (Fig. 2 and Fig. S2), a process we called MMEL. Interestingly, Wan et al. reported that upon pro-apoptotic BH3 signaling and pharmacological MOMP (mitochondrial outer membrane permeabilization) induction, endolysosomes increasingly contact mitochondria and then rapidly accumulate throughout the mitochondria^55^. In addition, Okuyama et al reported that Mieap under hypoxia can induce accumulation of lysosomes in mitochondria^41^. Thus, our results related to MMEL are consistent with the previous reports.

We also found that the STX17-SNAP29-VAMP7 complex contributes to mitochondria-lysosome contacts and MMEL (Figs. 4 and S6). It has been reported that endosomes can engulf mitochondria and deliver mitochondria to lysosomes for clearance^56^. On the other hand, mitochondria-derived vesicle (MDV) with a diameter of ~70–150 nm can be delivered directly to the lysosome/later endosome^25,33^. In yeast, mitochondria are in physical contact with vacuoles (lysosomes) via the vacuolar and mitochondrial patch (vCLAMP)^57^. The Chen lab reported that the mitochondria-associated protein Dosmit triggers mitochondrial enlargement and mediates the formation of double-membraned vesicles within enlarged mitochondria (megamitochondria) in Drosophila melanogaster^58^. Recently, the Nakamura lab also reported that mitolysosomes can enter mitochondria to perform mitochondrial recycling^59^. Thus, the mitochondrion has a number of ways to contact other organelles. Our results indicate that MMEL is a mode of mitochondria-lysosome contacts and mitochondrial processing that provides insight into understanding mitochondrial functions and mitochondria-lysosome crosstalk.

Mitochondria-lysosome crosstalk plays an important role in maintaining cellular homeostasis^20–23^. However, the underlying molecular mechanisms of mitochondria-lysosome crosstalk are still unclear. We found that under hypoxia, mitochondria-lysosome contacts increased significantly, and MMEL were also induced under hypoxia (Fig. 2). In addition, the STX17-SNAP29-VAMP7 complex may mediate megamitochondria-lysosome contacts and subsequent MMEL under hypoxia (Fig. 4). Moreover, hypoxia markedly increased mitochondria-lysosome contacts but not MMEL in *MFN1/2* DKD or *OPA1* knockdown cells (Fig. S3e and S3f), suggesting that mitochondrial fusion does not affect mitochondria-lysosome contacts. These data suggest that mitochondrial fusion cooperates with the STX17-SNAP29-VAMP7 complex to mediate MMEL. Therefore, only mitochondria-lysosome contacts cannot directly induce MMEL under hypoxia. First, megamitochondria must be formed, and then the lysosome contacts the megamitochondria. The contact force between lysosome and mitochondria may lead to invagination of megamitochondria, resulting in MMEL (Fig. 7i–b). It follows that both mitochondria-lysosome contacts and megamitochondria formation contribute to MMEL. It should be noted that the molecular mechanism of organelle-organelle contacts is complicated and additional proteins should be associated with the process of megamitochondria-lysosome contacts, fusion, and MMEL under hypoxia, which will be the focus of our future research.

Hypoxia induces autophagosome-dependent mitophagy through activation of the mitophagy receptors FUNDC1, BNIP3, and BNIP3L^29,30,60^. In addition, an alternative mitophagy pathway mediated by Ulk1/Rab9 protects the heart from ischemia^49^. Interestingly, we found that mitochondrial proteins were still significantly reduced under hypoxia in *ATG5* or *Rab9* depleted cells (Figs. 6d–g, and S9c–e). These findings suggest that there may be other pathways responsible for mitochondrial degradation in addition to mitophagy. In this framework, we found that hypoxia induces the MMEL process and lysosomal proteases are released into megamitochondria after MMEL (Figs. 2 and 5). In addition, hypoxia activates most mitochondrial proteases to degrade mitochondrial proteins (Fig. S11b–k). Taken together, both MMEL and activated mitochondrial proteases under hypoxia contribute to a mode of mitochondrial degradation, MSD (or mitoselfphagy). It should be noted that the area of megamitochondria (undergoing MSD) are larger than mitochondria undergoing mitophagy (Fig. S8g–j), but megamitochondria just partially degrade mitochondrial contents, and it is not excluded that megamitochondria can undergo mitochondrial fission to generate small mitochondria for mitophagy. Therefore, MSD and mitophagy corporately contribute to the degradation of mitochondria under hypoxia.

We describe here a mechanism of mitochondrial degradation, which we call MSD. MSD occurs under hypoxia and causes partial degradation of the mitochondria. Unlike mitophagy, it does not rely on the autophagy machinery. Both lysosomal and mitochondrial proteases are required for MSD. We propose that MSD is an important pathway to regulate mitochondrial quality under hypoxia and may be involved in human diseases under additional conditions.

## Methods

### Immunostaining, confocal microscopy analysis and imaging processing

Cells were grown on glass coverslips and then fixed in 4% paraformaldehyde at RT for 20 min and permeabilized with 0.1% Triton-X-100 for 10 min. Cells were blocked with phosphate-buffered saline (PBS) containing 10% fetal bovine serum (FBS) for 1 h at 4 °C and then incubated with primary antibodies in FBS for 1 h at RT. After washing, cells were incubated in the presence of secondary antibody conjugated to Alexa-488 (1:400), Cy3 (1:800), or Alexa-647 for 1 h at RT. After washing, coverslips were mounted in Cytifluor (Amersham Biosciences). Cells were analyzed and imaged using a Leica L8 or Zeiss LSM880 microscope with Airyscan. Laser excitation was performed with 488 nm, 561 nm, or 638 nm lasers.

Three-dimensional (3D) confocal imaging was performed using a ZEISS LSM880 microscope with Airyscan using a 63× oil immersion objective lens. Confocal z-slices of cells were acquired every 0.25 μm for a total of 5.0 μm. Images were captured using ZEN and reconstructed using 3D slice reconstruction. In addition, 3D surface reconstructions overlaid on the original data were analyzed and created using Imaris software.

For time-lapse imaging, optical z-sections of live cells stably expressing mito-GFP and LAMP1-mCherry were acquired using a Zeiss LSM880 microscope with Airyscan equipped with an environmental chamber set at 37 °C. Time-lapse images were acquired using ZEN and processed using the Airyscan automated processing algorithm in ZEN.

### Measurement of mitochondrial morphology and structure

Mitochondrial morphology and structure were first imaged using a confocal microscope or a transmission electron microscope (TEM) and then analyzed by ImageJ software. Mitochondrial morphology was determined according to previously described method^5^. Briefly, to determine mitochondrial morphology, at least 300 cells (10 mitochondria per cell) were randomly selected for quantitative analysis and visually scored into four classifications: ‘tubular’, ‘short tubular’, ‘fragmented’ and ‘large spherical (megamitochondria)’. To determine the size of mitochondria, TEM images were analyzed by ImageJ software to measure the area of mitochondria, and the relative size of mitochondria was provided.

### Measurement of mitochondria-lysosome contacts and MMEL

Cells were incubated in normoxia or hypoxia for the indicated times, then fixed and immunostained. 3D confocal imaging was performed using a microscope ZEISS LSM880 with Airyscan. The 3D images were analyzed and used for quantification of mitochondria-lysosome contacts or MMEL [all lysosomes (more than 500) from 10 cells were used for quantification in each experiment, at least 3 independent experiments]. Data were then analyzed and the number of mitochondria-lysosome contacts or MMEL per 100 lysosomes was shown (“per 10 cells” or “per 100 mitochondria” was not used because the number of lysosomes is significantly increased and mitochondrial morphology in each cell is dramatically altered in hypoxia). Colocalization of lysosomes with mitochondria was analyzed by ImageJ software. >5% and ≤50% of a lysosome colocalizing with a mitochondrion was scored into “mitochondria-lysosome contacts”, and >50% of a lysosome colocalizing with a mitochondrion was scored into “MMEL”. At least 3 independent experiments were performed to quantify MMEL. Statistical analysis was performed using Prism 8 (GraphPad Software).

### Transmission electron microscopy (TEM) analysis

Cells grown on 100 mm dishes were washed twice with PBS and fixed in 3% PFA, 0.5% glutaraldehyde, and 0.25% sucrose in 0.1 M sodium phosphate buffer (pH 7.4) for 1 h at RT. They were then washed with 0.1 M PB contain 0.25% sucrose (pH 7.4). Cells were then scratched, collected and compressed by gradient centrifugation. Samples were then postfixed with 2% osmium tetroxide in 0.1 M PB at 4 °C for 1 h, 2% uranyl acetate overnight 4 °C. Samples were dehydrated with different concentrations of ethanol, embedded in 812’s resin at room temperature for 4 h, and polymerized at 60 °C for 2 days. The blocks were ultrathin-sectioned at 70 nm with a diamond knife using an ultramicrotome (Leica). The sections were placed on copper grids and stained with 2% lead citrate at RT for 15 min and 2% uranyl acetate at RT for 10 min. Finally, the sections were processed according to standard electron microscopy procedures. Images were acquired using a JEM-1400Plus (JEOL) transmission electron microscope at an acceleration voltage of 100 kV.

### Quantification and statistical analysis

Quantitative data were obtained from Zeiss LSM 880 images with Airyscan and analyzed using ImageJ software. Data were compiled and processed using Microsoft Excel. Densitometry was performed using ImageJ software for quantitative analysis of bands on Western blottings and TEM. Statistical analysis was performed using Prism 8 (GraphPad software). Data were presented as mean ± SD or SEM, and statistical significance was determined by a one-tailed or two-tailed ANOVA or two-tailed *t*-test. *P* values are indicated in the figure. Source data are provided as a Source Data file.

### Statistics and reproducibility

For most in vitro experiments, at least three biologically independent samples were used for each experimental group; no power was calculated. No data were excluded from the analysis. Purposeful statistical subjects varied across experiments, and key findings were replicated at different times. No data were excluded from the analysis and the data distribution was assumed to be normal.

### Reporting summary

Further information on research design is available in the Nature Portfolio Reporting Summary linked to this article.

## Supplementary information

Supplementary Information

Description of Additional Supplementary Files

Supplementary Movie 1

Supplementary Movie 2

Supplementary Movie 3

Supplementary Movie 4

Supplementary Movie 5

Supplementary Movie 6

Supplementary Movie 7

Supplementary Movie 8

Supplementary Movie 9

Supplementary Movie 10

Supplementary Movie 11

Reporting Summary

## Data Availability

All data supporting the conclusions included in the paper are available within the paper and its supplementary information. All proteomics data generated in this study have been deposited in the PRIDE database under accession code PXD034375. Source data are provided with the paper. Source data are provided with this paper.

## Source data

Source Data
