# Supplementary material for: Hypoxia-reprogramed megamitochondrion contacts and engulfs lysosome to mediate mitochondrial self-digestion
Source: Nat Commun. 2023 Jul 11;14:4105. doi: 10.1038/s41467-023-39811-9 (PMC10336010; doi:10.1038/s41467-023-39811-9)
Supplement: Supplementary file 1 — Supplementary Information [file 41467_2023_39811_MOESM1_ESM.pdf]

SUPPLEMENTARY INFORMATION

MATERIALS AND METHODS

Cell Culture Reagents and Antibodies

HeLa, 293T, HCT116 and MEFs cells were cultured in Dulbecco's Modified Eagle Medium (DMEM) supplemented with 100 U/mL penicillin (Gibco), 10% (v:v) fetal bovine serum (Gibco), and 100 mg/mL streptomycin (Gibco) at 37°C with 5% (v:v) CO_2. Cells were maintained in either normoxia (21% O_2) or hypoxia (1% O_2) for the indicated time. Bafilomycin A1 (BafA1), DAPI, and Chloroquine (CQ) were purchased from Sigma-Aldrich. The following antibodies were used: anti-TOMM20 (Proteintech, 11802-1-AP), anti-LAMP1 (SantaCruz, sc-20011), anti-TIM23 (BD Biosciences, 611222), anti-COX2 (Abcam, ab15191), anti-HSP60 (SantaCruz, sc-13115), anti-LONP1 (Proteintech, 15440-1-AP), anti-COX4 (Proteintech, 11242-1-AP), anti-Drp1 (BD Biosciences, 611738), anti-Mff (SantaCruz, sc-398617), anti-CSTD (ABclonal, a13292), anti-ATG5 (ABclonal, a0203), anti-Mid49 (Proteintech, 16413-1-AP), anti-AFG3L2 (ABclonal, A15393), anti-OPA1 (BD Biosciences, 612607), anti-HIF-1α (BD Biosciences, 610909), anti-GFP (SantaCruz, sc-9996), anti-Yme1L (Proteintech, 11510-1-AP), anti-OMA1 (SantaCruz, H-11), anti-PARL (Proteintech, 26679-1-AP) anti-LAMP2 (Servicebio, GB11330), anti-Fis1 (Proteintech, 10956-1-AP), anti-Syntaxin17(Proteintech, 17815-1-AP), anti-HAX-1 (Proteintech, 11266-1-AP), anti-HTRA2 (Proteintech, 15775-1-AP), anti-SNAP29 (ABclonal, A4290), anti-VAMP7 (Proteintech, 22268-1-AP), anti-Actin (GNI, GNI4110-BA), anti-Tubulin (GNI, GNI4110-BT). The antibody against PGAM5 was a gift from Quan Chen. HRP-conjugated secondary antibodies were from Jackson ImmunoResearch Laboratories (31160). The secondary antibodies used for immunofluorescence were: donkey anti-mouse IgG Alexa Fluor-647 (Jackson, 715–605-150), donkey anti-mouse IgG Alexa Fluor-488 (Jackson), goat anti-rabbit IgG CY3 (Jackson, 111–165-003), goat anti-mouse IgG Alexa Fluor-594 (Yeasen, 33212ES60).

Plasmids and shRNAi Constructions

Homo LAMP1 cDNA was cloned into a modified pMSCV-puro (Addgene) constructs containing a C-terminal GFP or mCherry tag. Short hairpin mediated RNA interference (shRNAi) against MFN1, MFN2, OPA1, DRP1, FIS1, LONP1, LAMP2, Syntaxin-17, Rab9， BNIP3L, LAMP1, VAMP7, SNAP29, CTSB, or CTSD was performed using retroviral vector system as described previously [1]. For RNA interference, shRNA against target gene was

performed using a modified retroviral vector with the H1 promoter to drive the expression of shRNAs [2]. The target sequences for gene knockdown were designed according to the previous reports [1, 3, 4] or designed by siRNA Selection Program (Whitehead Institute for Biomedical Research). The target sequence with the best effect of knockdown to the certain gene were listed below.

Homo *ATG5*: 5′-CCTTTCATTCAGAAGCTGT -3′

Homo *LONP1*: 5′-GCACGTCATGGATGTTGTG -3′

Homo *OPA1*: 5′-GTTATCAGTCTGAGCCAGG -3′

Homo *Drp1*: 5′-GAGGTTATTGAACGACTCA -3′

Homo *LAMP2*: 5′- GCGGTCTTATGCATTGGAATT -3′

Homo *Mfn1*: 5′-GATACTAGCTACTGTGAAA -3′

Homo *Mfn2*: 5′-GGAAGAGCACCGTGATCAA -3′

Homo *Sytaxin 17*: 5′-GGTAGTTCTCAGAGTTTGAT-3′

Homo *Rab9*: 5'-CCGAGGATAGGTCAGATCA-3'

Homo *VAMP7*: 5'- GGAGAAAGATTGGAATTAT-3'

Homo *SNAP29*: 5'- AGACAGAAATTGAGGAGCA-3'

Homo *LAMP1*: 5'-CAGGGCAGATATAGATAAA-3'

Homo *BNIP3L*: 5'- AACACGTACCATCCTCATCCTTT-3'

Homo *CTSB*: 5'- GCTTGGAACTTCTGGACAA -3'

Homo *CTSD*: 5'- CCTCGTTTGACATCCACTA -3'

The shRNA empty vector was used as control.

Knockout (KO) Cell Lines Generation

Yme1L KO, *OMA1* KO, or *Yme1L* and *OMA1* double knockout (DKO) cell lines were generated by CRISPR/Cas9 gene editing. sgRNA sequences were designed using the MIT CRISPR design tool (http://crispr.mit.edu/), and cloned into the LentiCRISPR plasmid (Addgene 49535). Recombinant LentiCRISPR combining with plasmids VSV-G and psPAX2 were transfected into 293T cells to generate lentiviral particles, which were used to infect HCT-116 cells for gene knockout. After infection, the cells were selected with puromycin and sorted into 96-well dishes. After two weeks, the surviving clones were picked, expanded, and selected. Cell lines were further analyzed by Western blotting to confirm gene knockout.

The following guide sequences were used:

5'- ACATTAGCATCCACCTCACG -3' for human *OMA1*;
5'-GAGCTCTTCAAAGCATTTGC-3' for human *Yme1L*;

Western Blotting Analysis

Cells were collected by centrifugation, then were resuspended with 1xPBS and 2x sample buffer. After being boiled and centrifuged, the samples were subjected to SDS/PAGE and transferred to a PVDF membrane (Millipore, 0.45μm Shanghai, China). Two hours later, PVDF membranes were blocked by 5% non-fat milk for 1h, primary and secondary antibodies were then applied, and ECL (BioRad, Shanghai, China) substrates were added to the membrane and exposed by using the x-ray film.

Immunoelectron Microscopy

Immunoelectron microscopy was performed by chemical fixation and immunolabeling. General, HeLa cells overexpressing GFP-LAMP1 were fixed with 3 steps. Firstly, cells were treated with 4% paraformaldehyde (PFA) in PB buffer for 2 h at room temperature. Cells were treated with 0.01% saponin for 10 min and then closed with 0.1% fish gelatin for 30 min. Cells were then immunolabeled with anti-GFP and with 10 nm gold particles. Secondly, cells were treated with 2.5% glutaraldehyde in PB buffer for 10 min at room temperature. Thirdly, cells were then fixed with 1% osmium acid with 1.5% potassium ferricyanide for 30 min at room temperature. This was followed by step-by-step dehydration with ethanol and resin-impregnation followed by embedding. The resin was hardened and sliced onto copper nets by the Protein Research Technology Center of Tsinghua University. Samples were viewed on a JEM-1400Plus (JEOL) transmission electron microscope at an acceleration voltage of 100 kV.

Mito-Keima Mitophagy Assay

Mito-Keima mitophagy assay was performed as previously described [1]. Briefly, HeLa cells were infected with a lentivirus harboring the mito-Keima vector (a gift from Michael Lenard). Then, cells were treated without or with hypoxia in fresh growth medium for 24h, and subsequently analyzed by confocal microscopy. Living cells were cultured in new glass-bottom dishes. After treatment with hypoxia, cells were imaged and analyzed using a Leica SP8 confocal microscopy (63x oil objective NA 1.35) using an argon laser (448 nm, mito-Keima at neutral pH) and (552 nm, mito-Keima at acidic pH). Ratiometric (552 nm: 448 nm) analysis

was performed using ImageJ software.

Mitochondria Isolation and Liquid Chromatography Tandem Mass Spectrometry (LC-MS/MS) analysis

Mitochondria were isolated by the differential centrifugation and density gradient centrifugation. HeLa cells were treated with normoxia or hypoxia for 24 h, then the cells were collected. The collected cells were centrifuged at 400g for 5 min at 4°C, then 2ml buffer A (pH=7.2, 4°C; MOPS 10mM; Sucrose 83mM) was added to the precipitated cells and suspended. Then the samples were transferred into dounce homogenizer and dounced 100 to 120 times according to the amounts of cells. Then 2ml buffer B (pH=7.2, 4°C; MOPS 30mM; Sucrose 250mM) was added to mix, and the samples were transferred into EP tubes, and centrifuged at 1000g for 5 min at 4°C, the precipitation were discarded and the supernatant were further centrifuged at 12000g for 20 min at 4°C, and the precipitation was the fraction of crude mitochondria, and the supernatant was the fraction of cytosol. The fraction of crude mitochondria was further separated by sucrose density gradient centrifugation and obtain pure mitochondria (Sucrose solution concentration in polyallomer tube from top to bottom is 15%, 20%, 30%, 40%, 50%, 60%, 4ml per gradient, centrifuged at 18000g/min at 4°C for 90min). After sucrose gradient centrifugation, the sucrose concentration layer where mitochondria are located was absorbed, and then centrifuged at 20000g for 20 min at 4°C, and the precipitation was the fraction of pure mitochondria. The pure mitochondrial fraction was then solubilized with MT lysis buffer (Tris-HCL 10mM; Nacl 150mM; EDTA 2 mM; Triton X-100 0.2%; NP40 0.3%) for 30min at 4°C, then were centrifuged at 12000g for 20 min at 4°C, and the supernatant was the mitochondrial solution. The mitochondrial solution was then digested and analyzed by mass spectrometry. Nanoflow LC-MS/MS analysis of tryptic peptides was conducted on a Q Exactive HF Orbitrap coupled to an EASYnLC 1000 ultra-high-pressure system via anano-electrospray ionsource (all fromThermo FisherScientific).

Mass Spectrometry Data Analysis and Heatmap Production

The data of mass spectrometry were divided into four parts, which are outer mitochondrial membrane proteins, Intermembrane mitochondrial space and inter mitochondrial membrane proteins, mitochondrial matrix proteins, and oxidative phosphorylation complexes proteins. The data were classified, analyzed and processed for heatmap production. The data were

analyzed by Z-Score, and processed for heatmap production using MeV software. A Z-score is a numerical measurement that describes a value's relationship to the mean of a group of values. Z-score is measured in terms of standard deviations from the mean. If a Z-score is 0, it indicates that the data point's score is identical to the mean score. A Z-score of 1.0 would indicate a value that is one standard deviation from the mean. Z-scores may be positive or negative, with a positive value indicating the score is above the mean and a negative score indicating it is below the mean.

Quantitative real-time PCR analysis

RNA of cells was extracted using Trizol (Life Technologies, 15596-026) followed by DNase (Promega, M610A) treatment. And the cDNA was synthesized using the RevertAid Synthesis Kit (Thermo Scientific, K1622) according to the manufacturer's instructions. The cDNA samples were used as templates for quantitative real-time quantitative PCR (Q-RT-PCR) analysis using SYBR Green Supermix (ABclonal, RK21203) and the iCycler real-time PCR Detection System (Bio-Rad). The fold change of target mRNA expression was calculated using the 2-ΔΔCT method.

Focused ion beam/scanning electron microscopy (FIB-SEM) Analysis

HeLa or HCT116 cells were fixed and prepared as we have previously described [5]. Focused ion beam milling and SEM imaging were carried out using a FEI Helios NanoLab G3 UC (from Tsinghua, China). FIB milling was performed at 600 pA to 20 nA for the indicated samples. SEM-Imaging current was 0.4 nA. FIB milling steps were 10 nm/slice or 20 nm/slice and each slice was imaged. Accordingly, each image represents 500 nm of the stack, at 35k x magnification. The pixel size of the images was 2.19 nm. The selected mitochondrial images were reconstructed in 3D using Amira software, and the 3D representation of mitochondria was rendered by IMOD software.

Magic Red Assay

Magic Red^{TM} Cathepsin B kit (Bio-Rad, #937) was used to measure cathepsin B protease activity by detecting active cathepsins in whole, living cells. Magic Red reagent can be cleaved in the presence of cathepsins to generate a fluorescent product in live cells. HeLa cells stably

expressing mito-GFP were treated with normoxia or hypoxia for 24 hours, and then incubated with Magic Red reagent at 37 °C for 1 hour, followed by analysis and imaging by confocal microscopy. The number of mitochondria containing Magic Red fluorescence per 1000 mitochondria of 10 cells was then quantified for each experiment, and the experiment was repeated 3 times.

Mitochondrial ROS Measurement

Mitochondrial ROS (mtROS) was detected by MitoSOX^{TM} Red (Invitrogen) according to the manufacturer's instructions. Briefly, cells were incubated with 5uM MitoSOX with Hank's balanced salt solution at 37°C for 15min, then washed cells 3 times with HBSS solution (0.5mM $MgCl_2$, 1.26mM $CaCl_2$, 0.4mM $MgSO_4$, 5.33mM KCl, 0.44 KH_2PO_4, 4.16mM $NaHCO_3$, 138mM NaCl, 0.34mM Na_2HPO_4 and 5.56mM D-Glucose). The fluorescence was measured and imaged by confocal microscopy (excitation at 510nm, emission at 580nm). The fluorescence intensity was analyzed by ImageJ software.

Mitochondrial TMRM Measurement

HeLa overexpressing LAMP1-GFP cells were seeded on 30 mm confocal dishes and then treated with hypoxia for 24 h. Cells were then stained with 50 nM TMRM (Thermo Fisher Scientific) in complete medium for 30 min at 37°C. The stained cells were rinsed 3 times with pre-warmed HBSS and imaged in vivo in HBSS.

Bimolecular Fluorescence Complementation (BiFC)

BiFC [6] analysis was used to detect the contact or fusion between the mitochondrial membrane and the lysosomal membrane. Vectors expressing STX17-VC or SNAP29-VN was constructed. Then, HeLa cells expressing STX17-VC, SNAP29-VN, or STX17-VC plus SNAP29-VN were visualized and imaged by confocal microscopy.

High Sensitivity Structured Illumination Microscopy (HIS-SIM)

HIS-SIM analysis was performed as described previously [7]. Cells were seeded onto 30 mm confocal dishes and treated with hypoxia for 24 h, cells were then incubated with 250 nM PKMDR (Guangzhou Computational Super-resolution Biotech Co., Ltd.) for 15 min at 37°C, and then washed three times with PBS solution. The images were placed on a High Sensitivity

Structured Illumination Microscopy (HIS-SIM). All SIM images were analyzed using the Hessian-SIM microscopy method and ImageJ software.

High Pressure Freezing Samples Preparation

Cells were frozen under high pressure in the high-pressure freezer. Cells were quickly frozen and fixed with LEICA EM ICE and stored in liquid nitrogen. The fixative was prepared as follows: 1% osmium tetroxide (OsO_4) acetone solution was prepared and stored in liquid nitrogen. Three specimen holders carrying samples (three biological repeats) were combined in a standard 2.0 mL cryo-tube for the freeze substitution. Samples at liquid nitrogen temperature were transferred to fixative vials at an identical temperature and then placed in the Leica EM ASF2, where they were warmed to –90°C. Substitution was completed in 2.5 days and then the samples were warmed according to the following schedule: It takes 6h to heat up from -90°C to -60°C, 5°C/h, 8h at-60°C, and 6h to heat up from -60°C to -30°C 5°C/h, 8h at-30°C, and 6h to heat up from -30°C to 0°C, 4 h at 0°C, and 5h to heat up from 0°C to 25°C. Samples were rinsed two times in dried acetone. After epoxy propane gradient replacement, samples were embedded in 812 resin. Ultra-thin section preparation and subsequent transmission electron microscopic (TEM) observation were performed.

REFERENCES

1. Jian F*, et al.* Sam50 Regulates PINK1-Parkin-Mediated Mitophagy by Controlling PINK1 Stability and Mitochondrial Morphology. *Cell Rep* **23**, 2989-3005 (2018).
2. Chen H, Chomyn A, Chan DC. Disruption of fusion results in mitochondrial heterogeneity and dysfunction. *J Biol Chem* **280**, 26185-26192 (2005).
3. Yan C, Gong L, Chen L, Xu M, Abou-Hamdan H. PHB2 (prohibitin 2) promotes PINK1-PRKN/Parkin-dependent mitophagy by the PARL-PGAM5-PINK1 axis. *Autophagy* **16**, 419-434 (2020).
4. Zhang J*, et al.* Zinc oxide nanoparticles harness autophagy to induce cell death in lung epithelial cells. *Cell death Dis* **8**, e2954 (2017).
5. Ruan Y, Li H, Zhang K, Jian F, Tang J, Song Z. Loss of Yme1L perturbates mitochondrial dynamics. *Cell death Dis* **4**, e896 (2013).
6. Miller KE, Kim Y, Huh WK, Park HO. Bimolecular Fluorescence Complementation (BiFC) Analysis: Advances and Recent Applications for Genome-Wide Interaction Studies. *J Mol Biol* **427**, 2039-2055 (2015).
7. Chen L*, et al.* Loss of Sam50 in hepatocytes induces cardiolipin-dependent mitochondrial membrane remodeling to trigger mtDNA release and liver injury. *Hepatology* **76**, 1389-1408 (2022).

SUPPLEMENTARY FIGURES

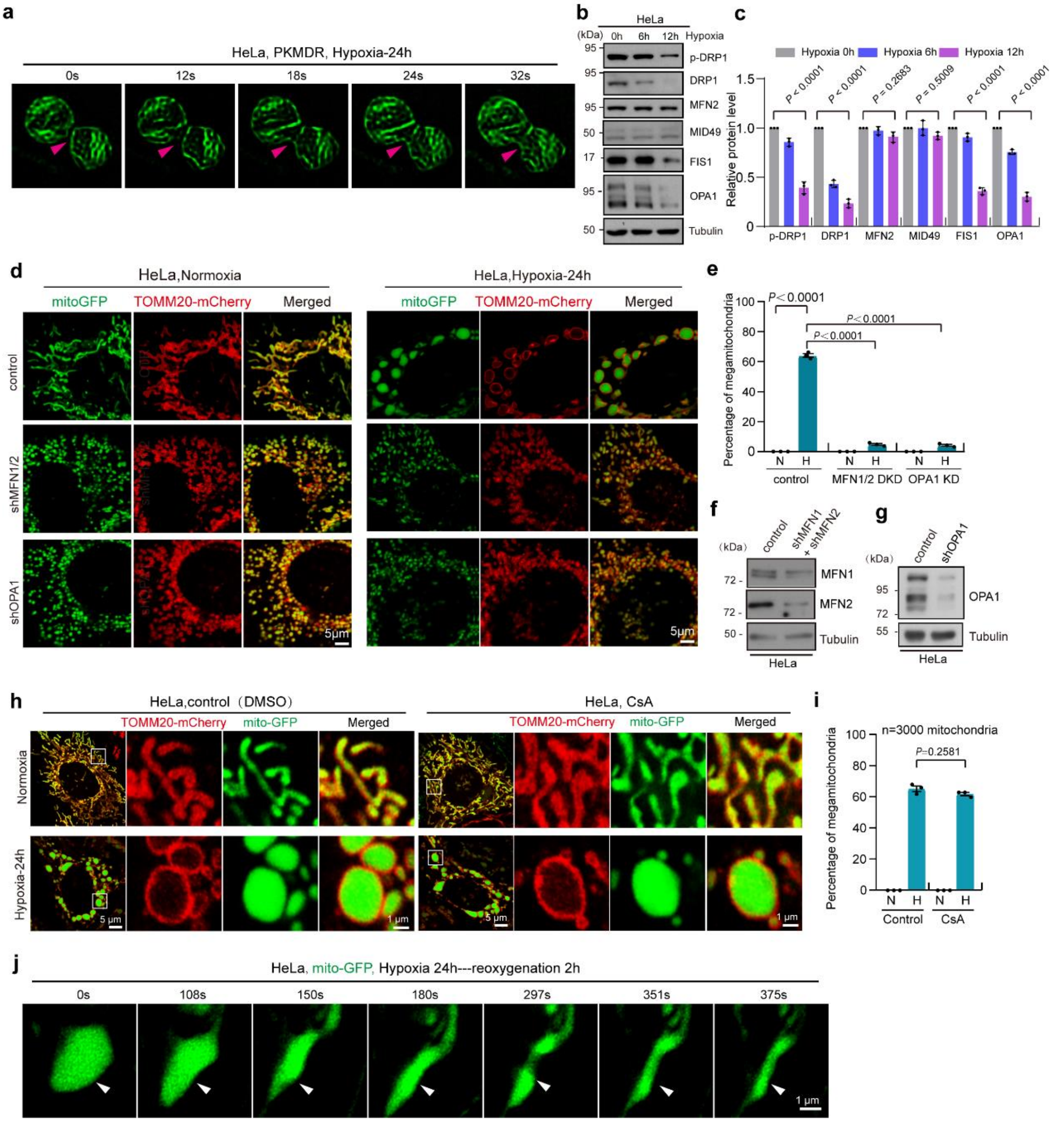

Figure S1. Mitochondrial fusion is required for the formation of megamitochondria under hypoxia

a HeLa cells were treated with hypoxia for 24h, and were directly stained with PKMDR (mitochondrial cristae and inner membrane stainer), and were analyzed by time-lapse confocal imaging with HIS-SIM (High Sensitivity Structured Illumination Microscope). **b-c** HeLa cells were exposed to hypoxia for 0h (normoxia), 6h, and 12h, respectively. Cell lysates were analyzed by Western blotting using antibodies against DRP1, p-DRP1, MFN2, MID49, FIS1, OPA1 or Tubulin (**b**). The relative protein levels were evaluated by densitometry analysis using

ImageJ software (**c**). Error bars represent means ± SEM, n = 3 independent experiments, and statistical significance were determined by a one-way ANOVA. **d**-**e** Control, *MFN1* and *MFN2* double knockdown (*MFN1/2* DKD), or *OPA1* knockdown HeLa cells stably co-expressing mito-GFP (mitochondria) and TOMM20-mCherry were exposed to normoxia or hypoxia for 24h, and were analyzed and imaged with confocal microscopy with Airyscan (**d**). Megamitochondria were quantified according to the criteria detailed in "Methods" (**e**). **f**-**g** Cell lysates of control, *Mfn1/2* double knockdown (**f**) or *OPA1* knockdown (**g**), HeLa cells were analyzed by Western blotting with the indicated antibodies. **h**-**i** HeLa cells stably expressing TOMM20-mCherry and mito-GFP (mitochondria) were exposed to normoxia or hypoxia in the presence of DMSO (control), or Cyclosporin A (CsA) for 24h. Mitochondrial morphology was visualized by confocal microscopy (**h**). Mitochondrial morphology was quantified (**i**) according to the criteria detailed in "Methods". **j** HeLa cells stably expressing mito-GFP (mitochondria) were treated with hypoxia for 24 hours followed by reoxygenation for 2 hours, and then were analyzed and imaged with confocal microscopy with Airyscan. Bars of **e** and **i** represent mean ± SEM, n = 3 independent experiments, statistical significance was assessed by a two-way ANOVA. *P*-values are indicated in the figure. Source data are provided as a Source Data file.

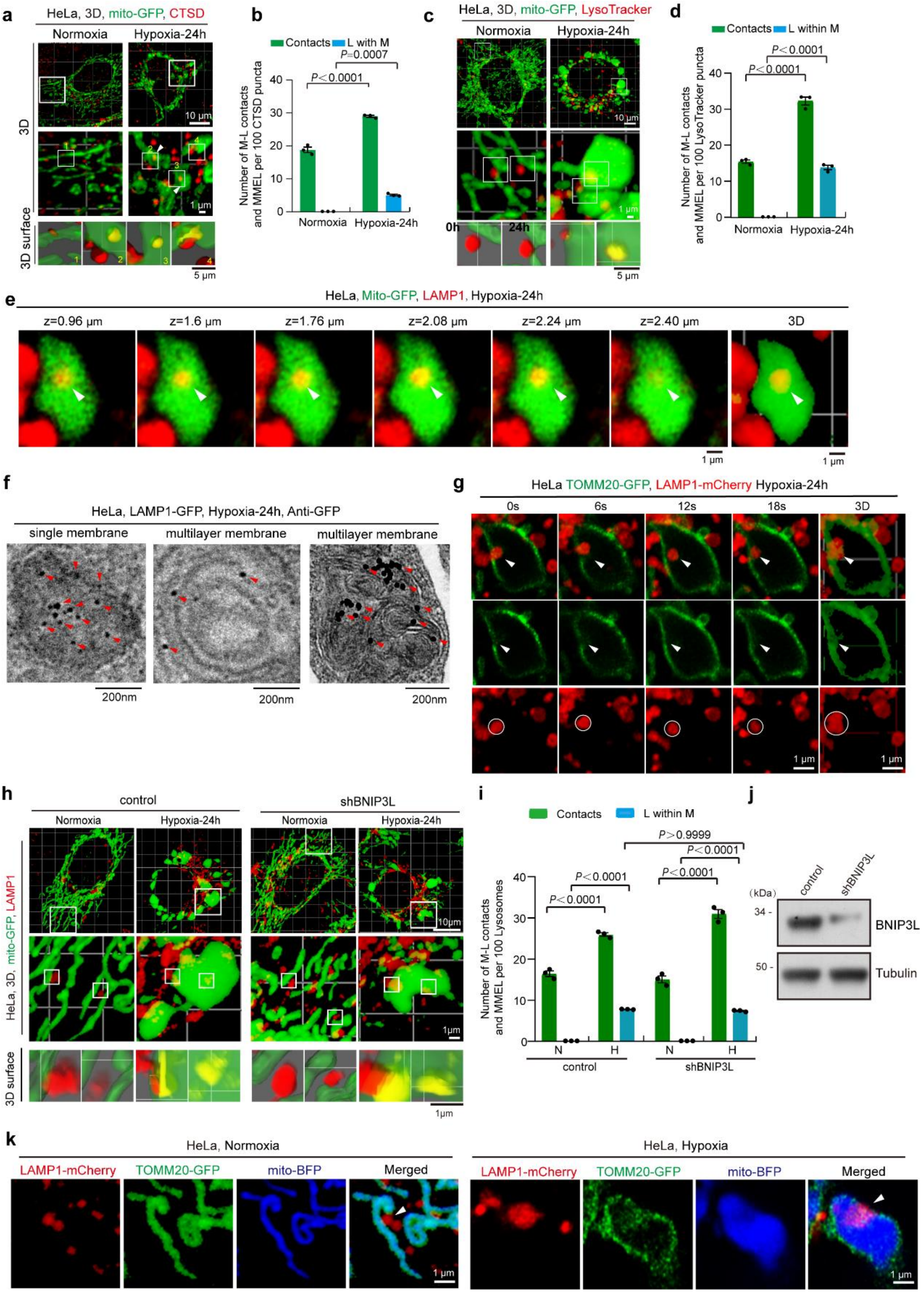

Figure S2. Hypoxia promotes lysosome entering into megamitochondria

a-d HeLa cells stably expressing mito-GFP (mitochondria) were treated with normoxia or hypoxia for 24h, and were immunostained with antibodies against lysosomal protease cathepsin

D (CTSD) (**a**) or stained with LysoTracker Red (**c**), and were analyzed by 3D imaging with confocal microscopy with Airyscan. Mitochondria (red) and lysosome (green) were displayed using 3D surface reconstructions (**a** and **c**). Mitochondria-lysosome contacts (contacts) and MMEL (engulfment) from 10 cells were quantified in each experiment, and the number of M-L contacts and MMEL per 100 lysosomes was displayed (**b** and **d**). **e** HeLa cells with expression of mito-GFP (mitochondria) were treated with hypoxia for 24h, and were immunostained with antibodies against LAMP1 (lysosome), then analyzed by 3D imaging with confocal microscopy with Airyscan. Representative images of confocal Z-stacks were shown. White arrowhead: lysosome. **f** HeLa cells expressing LAMP1-GFP cells were treated with hypoxia for 24h, and then were immunostained for GFP immuno-gold staining and immunoelectron microscopy analysis. **g** HeLa cells expressing LAMP1-mCherry (lysosomes) and TOMM20-GFP (mitochondria) were treated with hypoxia for 24h, and then tracked by time-lapse confocal imaging. "L": lysosome, "M": mitochondrion. **h-j** Control or *BNIP3L* knockdown (sh*BNIP3L*) HeLa cells stably expressing mito-GFP (mitochondria) were treated with normoxia or hypoxia for 24h. Cells were immunostained with an antibody against LAMP1 (lysosome) and analyzed by 3D imaging with confocal microscopy with Airyscan. Mitochondria (green) and lysosome (red) were displayed using 3D surface reconstructions (**h**). Mitochondria-lysosome contacts and MMEL from 10 cells were quantified in each experiment, and the number of M-L contacts and MMEL per 100 lysosomes was displayed (**i**). Cell lysates of control, *BNIP3L* knockdown HeLa cells were analyzed by Western blotting with the indicated antibodies (**j**). **k** HeLa cells expressing LAMP1-mCherry, TOMM20-GFP and mito-BFP were treated with normoxia or hypoxia for 24h, and analyzed by confocal microscopy. Representative images were shown, and the white arrowhead indicates lysosome. Bars of **b**, **d** and **i** represent mean ± SEM, n = 3 independent experiments, statistical significance was assessed by a two-way ANOVA. *P*-values are indicated in the figure. Source data are provided as a Source Data file.

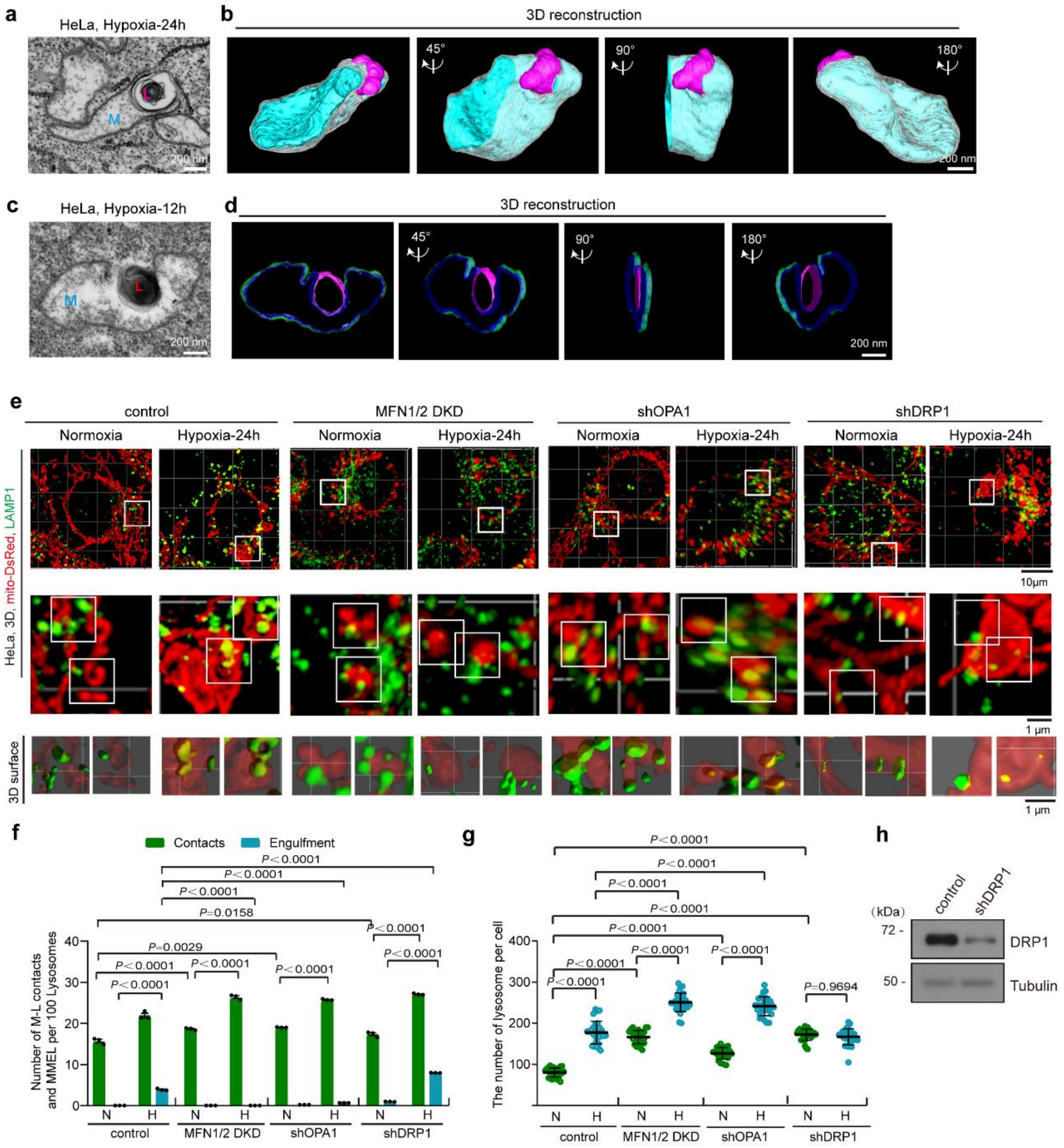

Figure S3. Mitochondrial fusion is required for MMEL

a-b The 70nm section of the specimen of HeLa cells exposed to hypoxia (24h) was analyzed and imaged by Fei Tecnai Spirit Transmission Microscope. 3D reconstruction and segmentation of mitochondrion and lysosome were performed using 3D-IMOD software. Green, mitochondrial outer membrane; Blue, mitochondrial inner boundary membrane; Purple-red, lysosome. **c-d** HeLa cells exposed to hypoxia (12h) were analyzed by FIB-SEM, and representative thin-section FIB-SEM images showing mitochondria-lysosome contacts were displayed (**c**). One section of the specimen is 10 nm thick (70 nm thick in total). 3D reconstruction and segmentation of mitochondrion and lysosome from FIB-SEM images was

performed using 3D-IMOD software (**d**). “M” indicates mitochondrion, “L” indicates lysosome. Purple red, lysosome; White, mitochondrial outer membrane; Cyan, mitochondrial inner boundary membrane. **e-f** Control, *MFN1/2* double knockdown (*MFN1/2* DKD, sh*MFN1* plus sh*MFN2*), *OPA1* knockdown (sh*OPA1*), or *DRP1* knockdown (sh*DRP1*) HeLa cells stably expressing mito-DsRed (mitochondria) were treated with normoxia or hypoxia for 24h. Cells were then fixed, immunostained with anti-LAMP1 (lysosome) antibody, and analyzed by 3D imaging with confocal microscopy with Airyscan. Mitochondria (red) and lysosome (green) were displayed using 3D surface reconstructions overlaid upon original data with Imaris software (**e**). The middle images show enlargements of the boxed areas in the top images, and the bottom images are 3D surface reconstructions of the middle images. Mitochondria-lysosome contacts (contacts) and MMEL (engulfment) from 10 cells were quantified in each experiment, and the number of M-L contacts and MMEL per 100 lysosomes was displayed (**f**). Bars of represent mean ± SEM, n = 3 independent experiments, statistical significance was assessed by a two-way ANOVA. **g** Control, *MFN1/2* DKD, sh*OPA1*, or sh*DRP1* HeLa cells were treated with normoxia or hypoxia for 24h, the number of lysosomes per cell were then quantified. n=30 cells. Data with error bars are presented as mean ± SD, statistical significance was assessed by a two-way ANOVA. **h** Cell lysates of control, *DRP1* knockdown HeLa cells were analyzed by Western blotting with the indicated antibodies. *P*-values are indicated in the figure. Source data are provided as a Source Data file.

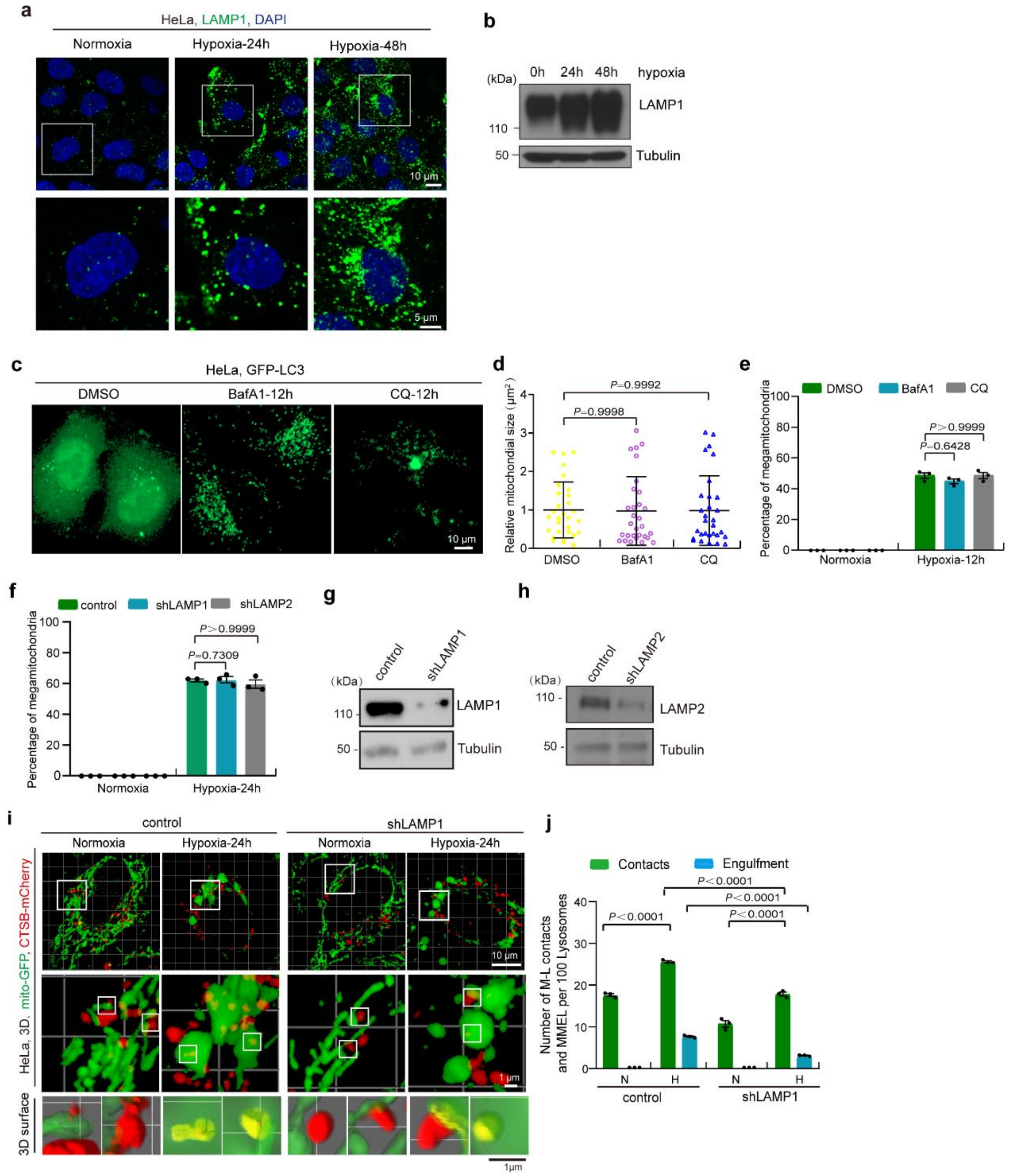

Figure S4. The effect of hypoxia on lysosomal biogenesis and mitochondrial size

a-b HeLa cells were treated with normoxia or hypoxia for 24h and 48h, and were immunostained with anti-LAMP1 antibodies and stained with DAPI, and then were analyzed by confocal microscopy (**a**). Cell lysates were analyzed by Western blotting with the indicated antibodies (**b**). **c** HeLa cells stably expressing GFP-LC3 were treated with normoxia or hypoxia in the presence of DMSO, Bafilomycin A1 (BafA1), or chloroquine (CQ) for 12h. Cells were then analyzed and imaged by confocal microscopy with Airyscan. **d-e** HeLa cells were treated with normoxia or hypoxia in the presence of DMSO, Bafilomycin A1 (BafA1), or chloroquine

(CQ) for 24h. Mitochondrial morphology was visualized by confocal microscope. Mitochondrial size was analyzed by ImageJ software (**d**), and megamitochondria were quantified (**e**) according to the criteria detailed in “Methods”. Bars of represent mean ± SD, n = 3 independent experiments, statistical significance was assessed by a one-way ANOVA. **f-h** Control, *LAMP1* knockdown (sh*LAMP1*) or *LAMP2* knockdown (sh*LAMP2*) HeLa cells were treated with normoxia or hypoxia for 24h. Then mitochondrial morphology was visualized by confocal microscope. Mitochondrial morphology was quantified (**f**) according to the criteria detailed in “Methods”. Cell lysates were analyzed by Western blotting with anti-LAMP1 (**g**), anti-LAMP2 (**h**) or anti-Tubulin antibodies. **i-j** Control or *LAMP1* knockdown (sh*LAMP1*) HeLa cells stably co-expressing mitoGFP (mitochondria) and CTSB-mCherry were treated with normoxia or hypoxia for 24h. Cells were analyzed by 3D imaging with confocal microscopy with Airyscan. Mitochondria (green) and lysosome (red) were displayed using 3D surface reconstructions overlaid upon original data with Imaris software (**i**). The middle images show enlargements of the boxed areas in the top images, and the bottom images are 3D surface reconstructions of the middle images. Mitochondria-lysosome contacts and MMEL from 10 cells were quantified in each experiment, and the number of M-L contacts and MMEL per 100 lysosomes was displayed (**j**). Bars of **f** and **j** represent mean ± SEM, n = 3 independent experiments, statistical significance was assessed by a two-way ANOVA. *P*-values are indicated in the figure. Source data are provided as a Source Data file.

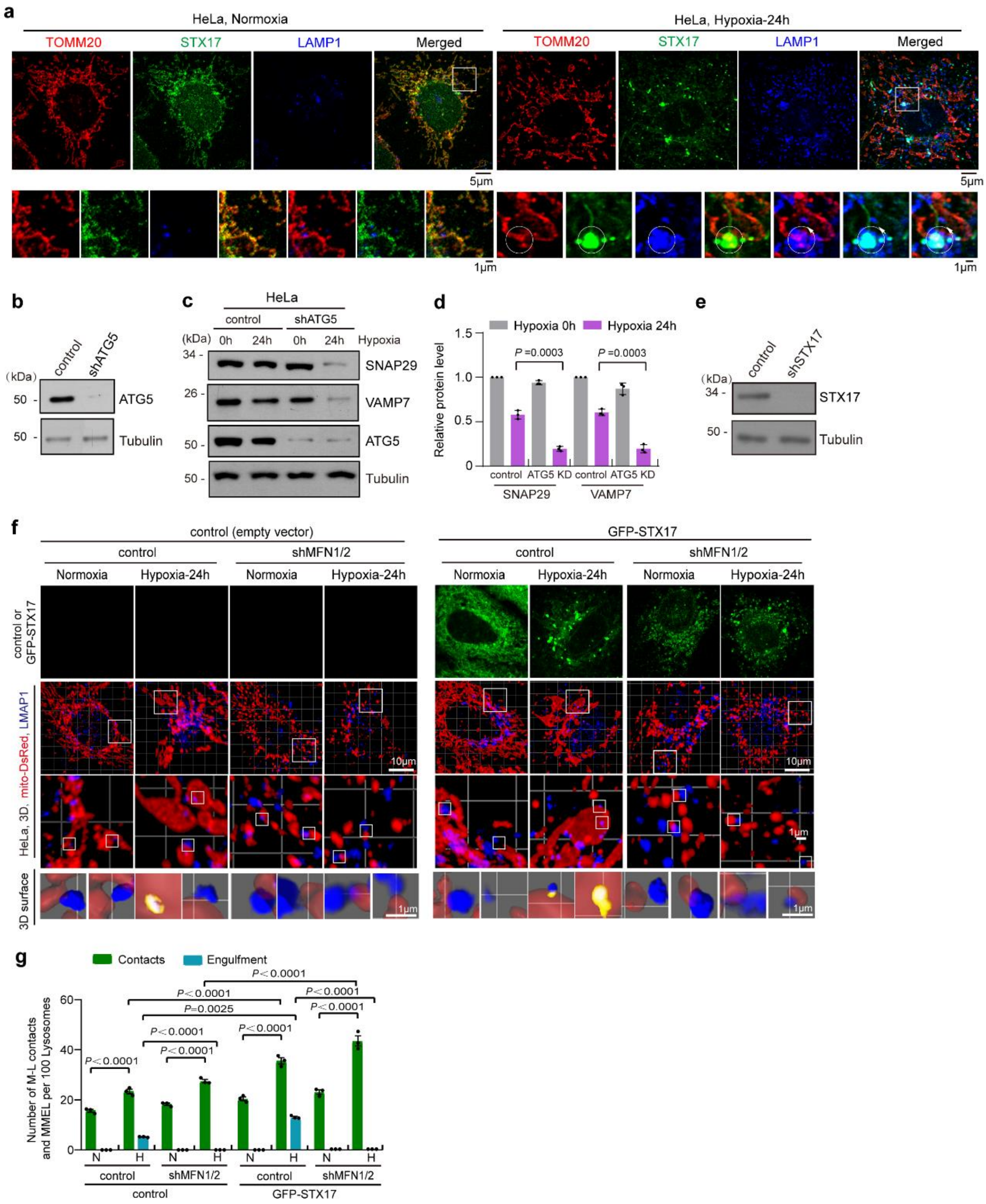

Figure S5. Mitochondria partially fuse with lysosome under hypoxia

a HeLa cells were exposed to normoxia or hypoxia for 24h, and were immunostained with antibodies against TOMM20 (mitochondria, red), LAMP1 (lysosome, blue), or STX17 (green) and were analyzed by confocal microscopy with Airyscan. **b** Cell lysates of control, *ATG5* knockdown HeLa cells were analyzed by Western blotting with the indicated antibodies. **c-d** Control or *ATG5* knockdown HeLa cells were exposed to hypoxia for 0h (normoxia), 24h. Cell

lysates were then analyzed by Western blotting with the indicated antibodies (**c**). The relative protein levels were further evaluated by densitometry analysis using ImageJ software (**d**). Error bars indicate the mean ± SD of the experiments, n = 3 independent experiments, statistical significance was assessed by two-tailed *t*-test. **e** Cell lysates of control, *STX17* knockdown HeLa cells were analyzed by Western blotting with the indicated antibodies. **f-g** Control or *MFN1/2* double knockdown (sh*MFN1/2*) HeLa cells with or without expression of GFP-STX17 (green) were exposed to normoxia or hypoxia for 24h. Cells were then fixed, immunostained with anti-LAMP1 (lysosome, blue) or anti-TOMM20 (mitochondria, red) antibody, and analyzed by 3D imaging with confocal microscopy with Airyscan. Mitochondria (red) and lysosome (blue) were displayed using 3D surface reconstructions overlaid upon original data with Imaris software (**f**). Mitochondria-lysosome contacts (contacts) and MMEL (engulfment) from 10 cells were quantified in each experiment, and the number of M-L contacts and MMEL per 100 lysosomes was displayed (**g**). Error bars indicate the mean ± SEM of the experiments, n = 3 independent experiments, statistical significance was assessed by a two-way ANOVA. *P*-values are indicated in the figure. Source data are provided as a Source Data file.

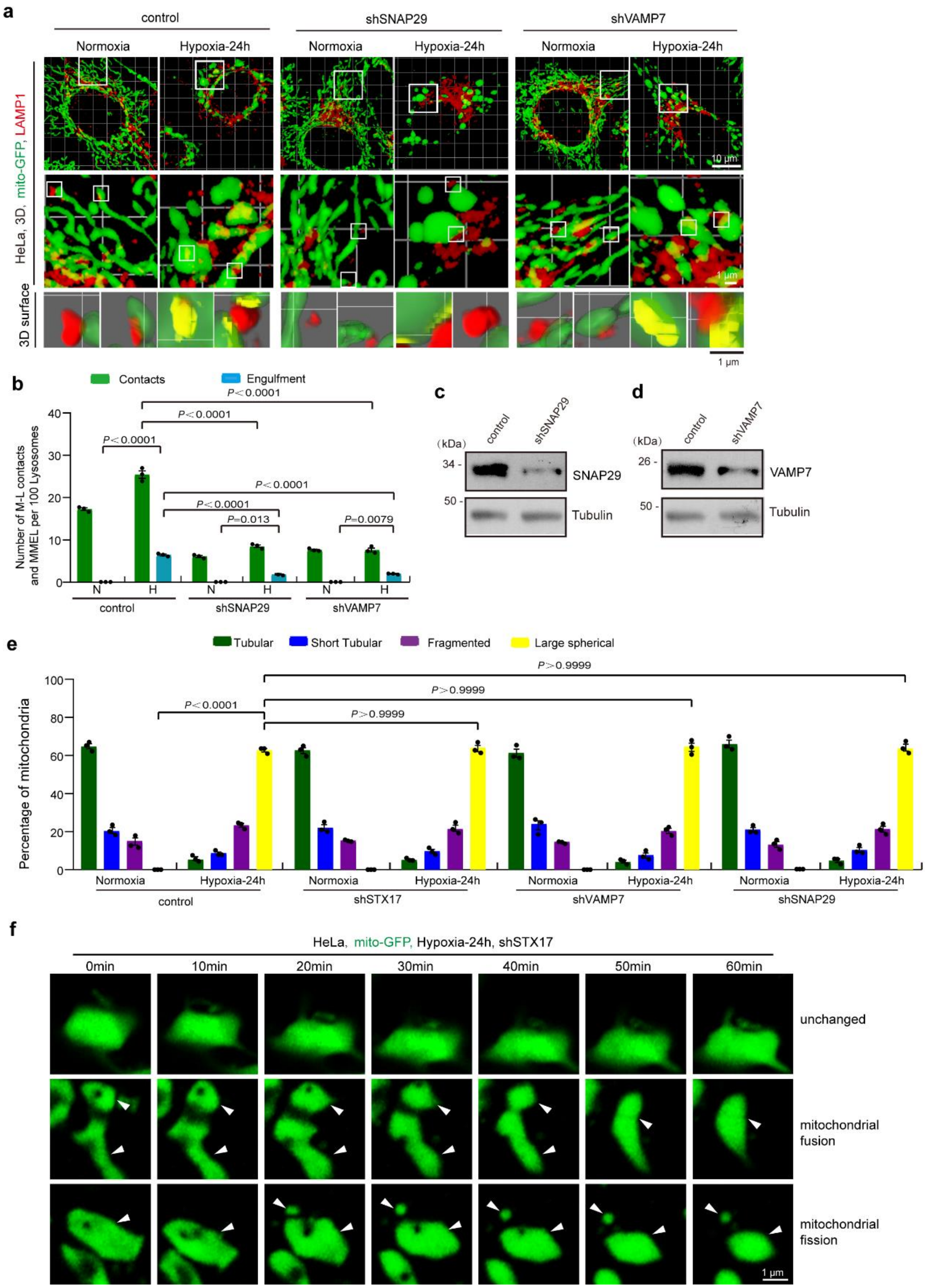

Figure S6. Hypoxia induces a new pathway of mitochondrial degradation

a-d Control, *VAMP7*, or *SNAP29* knockdown (control, sh*VAMP7*, or sh*SNAP29*) HeLa cells stably expressing mito-GFP (mitochondria) were treated with normoxia or hypoxia for 24h.

Cells were then fixed and which were immunostained with an antibody against LAMP1 (lysosome) and analyzed by 3D imaging with confocal microscopy with Airyscan. Mitochondria (green) and lysosome (red) were displayed using 3D surface reconstructions overlaid upon original data with Imaris software (**a**). Mitochondria-lysosome contacts (contacts) and MMEL (engulfment) from 10 cells were quantified in each experiment, and the number of M-L contacts and MMEL per 100 lysosomes was displayed (**b**). Error bars indicate the mean ± SEM of the experiments, n = 3 independent experiments, statistical significance was assessed by a two-way ANOVA. Cell lysates of control, *SNAP29* knockdown (**c**), or *VAMP7* knockdown (**d**) HeLa cells were analyzed by Western blotting with the indicated antibodies. **e** Control, *VAMP7*, or *SNAP29* knockdown HeLa cells were treated with normoxia or hypoxia for 24h. Cells were then fixed and immunostained with anti-TOMM20 antibody. Mitochondrial morphology was visualized by confocal microscopy. Mitochondrial morphology was quantified according to the criteria detailed in "Methods". Error bars indicate the mean ± SEM of the experiments, n = 3 independent experiments, statistical significance was assessed by a two-way ANOVA, statistical significance was assessed by a two-way ANOVA. **f** *STX17* knockdown HeLa cells stably expressing mito-GFP (mitochondria) were treated with hypoxia for 24 hours, and living cells were tracked and imaged with confocal microscopy with Airyscan. Mitochondrial fusion, fission, or unchanged dynamic events were displayed. *P*-values are indicated in the figure. Source data are provided as a Source Data file.

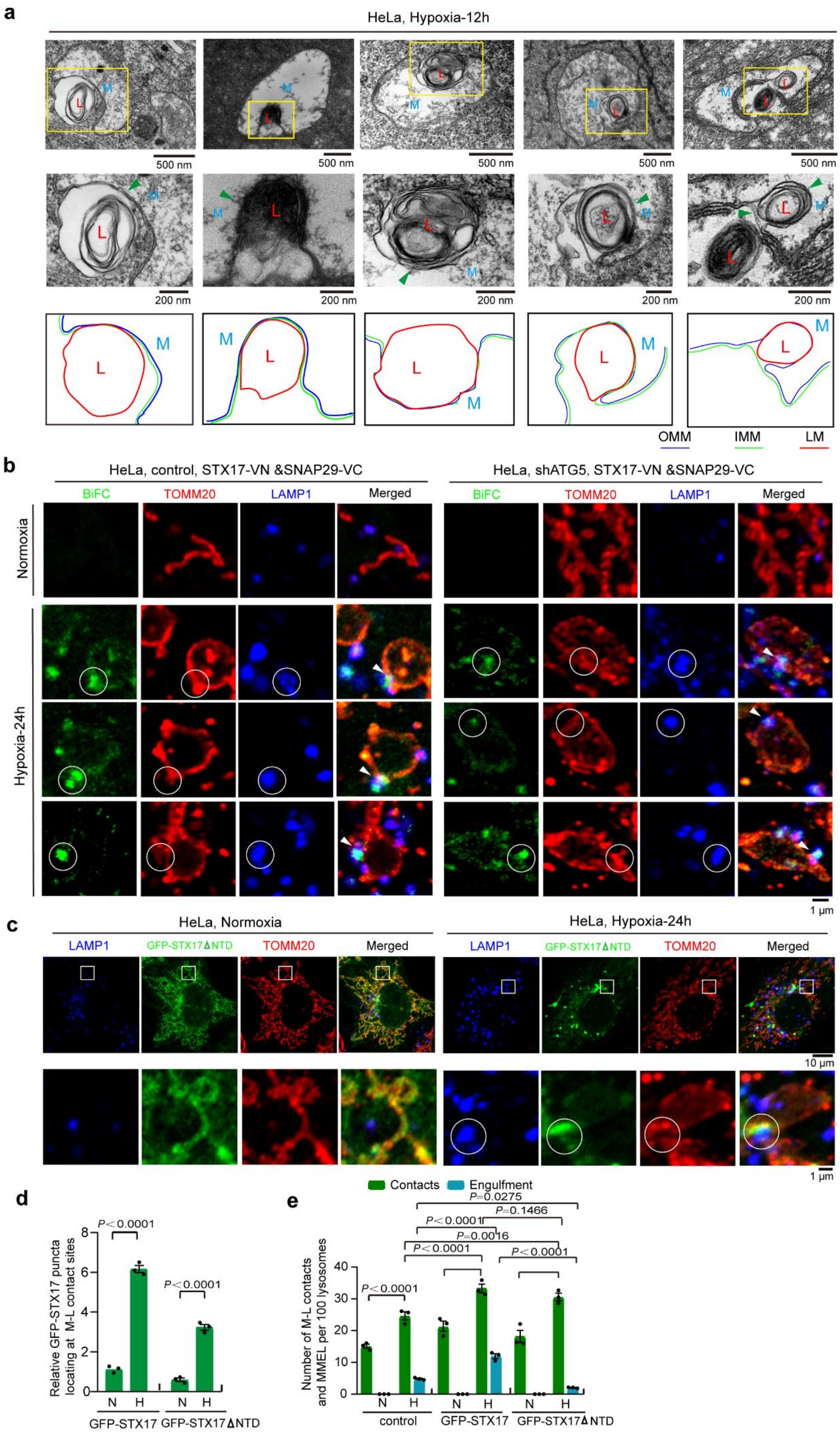
a
HeLa, Hypoxia-12h
L
M
500 nm
200 nm
OMM
IMM
LM
b
HeLa, control, STX17-VN &SNAP29-VC
HeLa, shATG5, STX17-VN &SNAP29-VC
BiFC
TOMM20
LAMP1
Merged
Normoxia
Hypoxia-24h
1 μm
c
HeLa, Normoxia
HeLa, Hypoxia-24h
LAMP1
GFP-STX17ΔNTD
TOMM20
Merged
10 μm
1 μm
d
Relative GFP-STX17 puncta locating at M-L contact sites
P < 0.0001
P < 0.0001
N
H
GFP-STX17
GFP-STX17ΔNTD
e
Contacts
Engulfment
Number of M-L contacts and MMEL per 100 lysosomes
P=0.0275
P=0.1466
P < 0.0001
P=0.0016
P < 0.0001
P < 0.0001
P < 0.0001
control
GFP-STX17
GFP-STX17ΔNTD

Figure S7. Mitochondria partially fuse with lysosome under hypoxia

a Hela cells were treated with hypoxia for 12h, and cell samples were then processed and analyzed by TEM. Representative TEM images display merge (fusion) of the mitochondrial membrane with the lysosomal membrane. The middle images show enlargements of the boxed areas in the top images. The red "L" indicates lysosome and the bule "M" indicates mitochondrion. The bottom images are depictions of mitochondrial and lysosomal membranes in the middle images. "OMM" indicates outer mitochondrial membrane, "IMM" represents inner mitochondrial membrane, and "LM" indicates lysosomal membrane. **b** Control or *ATG5* knockdown (sh*ATG5*) HeLa cells were co-transfected with STX17-VN and SNAP29-VC. 12 hours later, cells were treated with normoxia or hypoxia for 24h, and then fixed, immunostained with anti-LAMP1 (lysosome) antibody and anti-Tom20 (mitochondria) antibody, and analyzed by confocal microscopy. **c-e** HeLa cells were transiently transfected with plasmids expressing the GFP-STX17 or GFP-STX17 ΔNTD (deletion of STX17 N terminal transmembrane domain). 12 hours later, cells were exposed to normoxia or hypoxia for 24h, then immunostained with anti-LAMP1 (lysosome) antibody and anti-TOM20 (mitochondria) antibody, and analyzed by confocal microscopy (**c**). The number of GFP-STX17 puncta locating at megamitochondria-lysosome contact sites per cell was quantified (**d**), n=10 cells for each experiment. In addition, mitochondria-lysosome contacts (contacts) and MMEL (engulfment) from 10 cells were quantified in each experiment, and the number of M-L contacts and MMEL per 100 lysosomes was displayed (**e**). Error bars indicate the mean ± SEM of the experiments, n = 3 independent experiments, statistical significance was assessed by a two-way ANOVA. *P*-values are indicated in the figure. Source data are provided as a Source Data file.

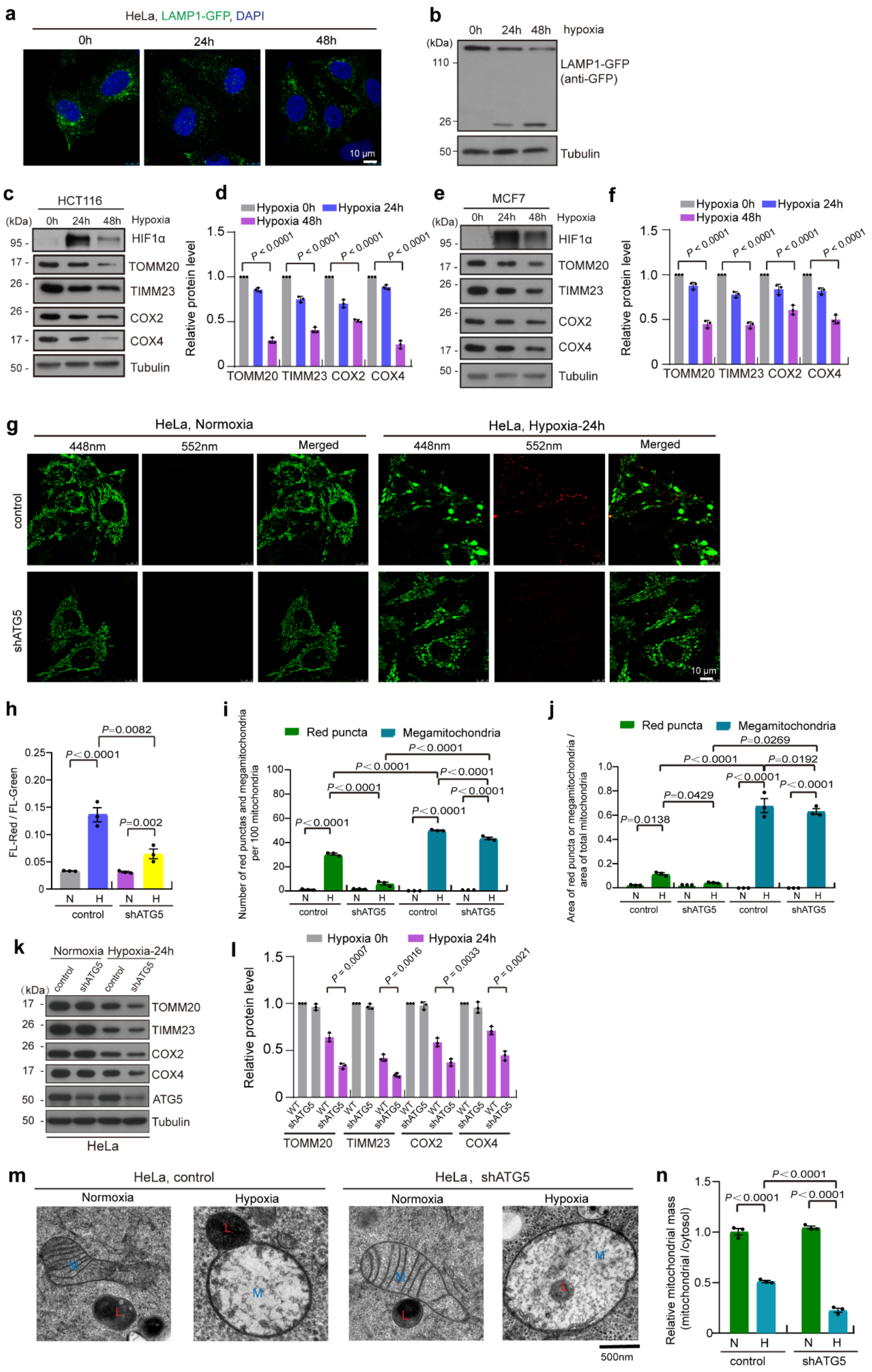

a
HeLa, LAMP1-GFP, DAPI
0h
24h
48h
10 μm
b
0h 24h 48h hypoxia
(kDa)
110
26
50
LAMP1-GFP
(anti-GFP)
Tubulin
c
HCT116
(kDa) 0h 24h 48h Hypoxia
HIF1α
TOMM20
TIMM23
COX2
COX4
Tubulin
d
Hypoxia 0h
Hypoxia 24h
Hypoxia 48h
Relative protein level
P < 0.0001
TOMM20 TIMM23 COX2 COX4
e
MCF7
f
g
HeLa, Normoxia
HeLa, Hypoxia-24h
448nm
552nm
Merged
control
shATG5
h
FL-Red / FL-Green
P=0.0082
P<0.0001
P=0.002
N H N H
control shATG5
i
Red puncta
Megamitochondria
Number of red punctas and megamitochondria per 100 mitochondria
P<0.0001
j
Area of red puncta or megamitochondria / area of total mitochondria
P=0.0269
P=0.0192
P=0.0429
P=0.0138
k
Normoxia Hypoxia-24h
control shATG5 control shATG5
ATG5
HeLa
l
Hypoxia 0h
Hypoxia 24h
P = 0.0007
P = 0.0016
P = 0.0033
P = 0.0021
WT
shATG5
m
HeLa, control
HeLa, shATG5
Normoxia
Hypoxia
500nm
n
Relative mitochondrial mass (mitochondrial /cytosol)

Figure S8. Hypoxia induces a new pathway of mitochondrial degradation

a-b HeLa cells expressing LAMP1-GFP were treated with hypoxia for 0h (normoxia), 24h, and 48h, and analyzed by confocal microscope (**a**). Cell lysates were analyzed by Western blotting with the indicated antibodies (**b**). **c-f** HCT116 (**c**) or MCF7 (**e**) cells were exposed to hypoxia for 0h (normoxia), 24h, and 48h. Cell lysates were analyzed by Western blotting with the indicated antibodies. The relative protein levels were further evaluated by densitometry analysis using ImageJ software (**d** and **f**). Error bars represent means ± SD of the experiments, n = 3 independent experiments and statistical significance were determined by a one-way ANOVA. **g-j** Control or *ATG5* knockdown (sh*ATG5*) HeLa cells expressing mito-Keima were exposed to normoxia or hypoxia for 24h. Living cells were then imaged for mito-Keima with 458 nm and 561 nm laser excitation by confocal microscope (**g**). The ratio of red (FL-red) to green (FL-green) fluorescence was quantified by ImageJ software (**h**). The red mitochondria (indicating mitochondria undergoing mitophagy) or megamitochondria were counted (**i**). The ratio of the area of red mitochondria or megamitochondria to the area of total mitochondria was quantified by ImageJ software (**j**). n=10 cells for each experiment. **k-l** Control or *ATG5* knockdown (sh*ATG5*) HeLa cells were exposed to normoxia or hypoxia (24h). Cell lysates were then analyzed by Western blotting with the indicated antibodies (**k**). The relative protein levels were further evaluated by densitometry analysis using ImageJ software (**l**). Error bars indicate the mean ± SD of the experiments, n = 3 independent experiments, statistical significance was assessed by two-tailed *t-test*. **m-n** Control or sh*ATG5* HeLa cells treated with normoxia or hypoxia (24h) and then were fixed by high pressure freezing process and analyzed by TEM (**m**). The ratio of mitochondrial or cytosolic electron density represents the relative mitochondrial mass (**n**). n=30 mitochondria for each experiment. Bars of **i, j** and **n** represent mean ± SEM of of the experiments, n = 3 independent experiments, statistical significance was assessed by a two-way ANOVA. *P*-values are indicated in the figure. Source data are provided as a Source Data file.

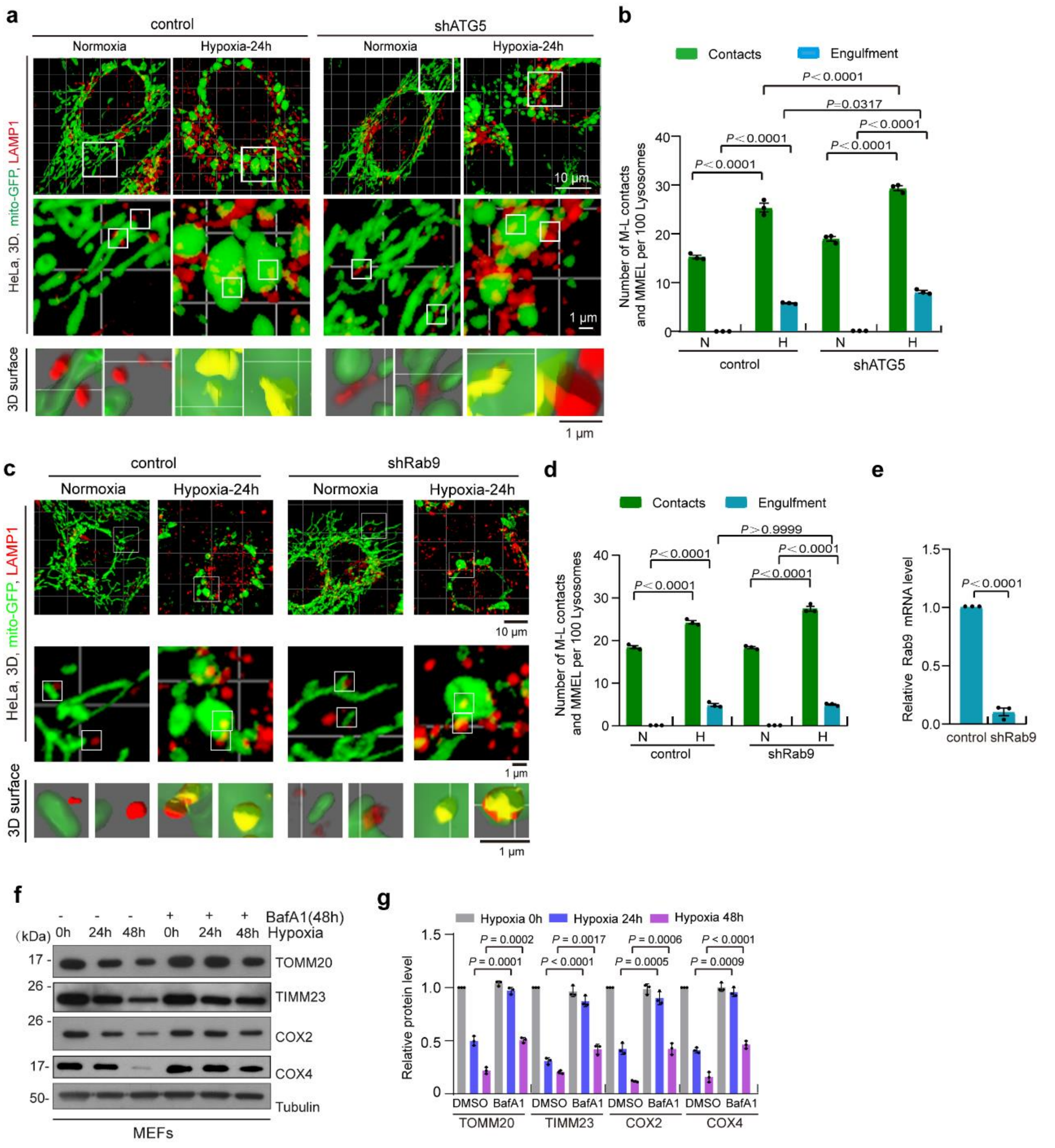

Figure S9. ATG5- or Rab9-mediated mitophagy has no effect on MMEL

a-d Control or *ATG5* knockdown HeLa cells stably expressing mito-GFP (mitochondria) were treated with normoxia or hypoxia for 24h. Control or *Rab9* knockdown (sh*Rab9*) HeLa cells stably expressing mito-GFP (mitochondria) were treated with normoxia or hypoxia for 24h. Cells were then fixed and which were immunostained with an antibody against LAMP1 (lysosome) and analyzed by 3D imaging with confocal microscopy with Airyscan. Mitochondria (green) and lysosome (red) were displayed using 3D surface reconstructions overlaid upon original data with Imaris software (**a**, **c**). The middle images show enlargements of the boxed areas in the top images, and the bottom images are 3D surface reconstructions of

the middle images. Mitochondria-lysosome (contacts) and MMEL (engulfment) from 10 cells were quantified in each experiment, and the number of M-L contacts and MMEL per 100 lysosomes was displayed (**b**, **d**). Error bars indicate the mean ± SEM of the experiments, n = 3 independent experiments, statistical significance was assessed by a two-way ANOVA. **e** Control or *Rab9* knockdown (sh*Rab9*) HeLa cells were extracted, and the relative levels of Rab9 mRNA were analyzed by quantitative RT-PCR. Bars of represent mean ± SEM, n = 3 independent experiments, statistical significance was assessed by two-tailed *t*-test. **f-g** MEFs were treated with hypoxia for the indicated time in the presence or absence of bafilomycin A1 (BafA1, 200nM). Cell lysates were then analyzed by Western blotting with anti-TOMM20, anti-TIMM23, anti-COX2, anti-COX4, or anti-Tubulin antibodies (**f**). The relative protein levels were further evaluated by densitometry analysis using ImageJ software (**g**). Error bars indicate the mean ± SD of the experiments, n = 3 independent experiments, statistical significance was assessed by two-tailed *t*-test. *P*-values are indicated in the figure. Source data are provided as a Source Data file.

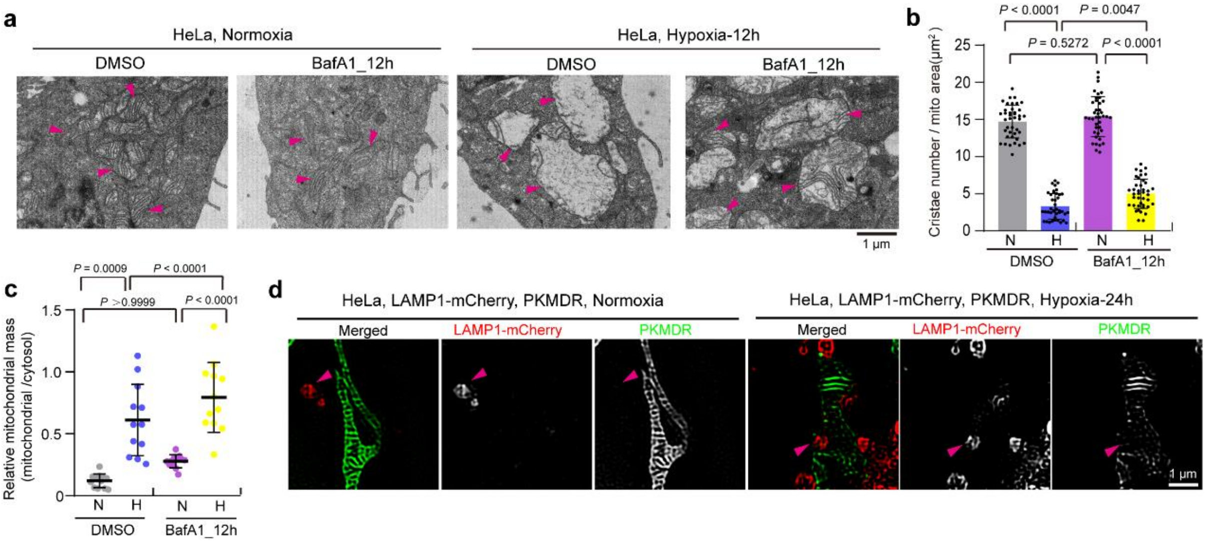

Figure S10. The effect of hypoxia on mitochondrial ultrastructure

a-c HeLa cells were treated with normoxia or hypoxia in the presence of DMSO (control), or BafA1 for 12h. Cell samples were then analyzed by TEM. Representative TEM images containing mitochondria were displayed (**a**), and the red arrowhead indicates mitochondrion. The number of mitochondrial cristae per mitochondrial area (1 μm^2) was then quantified by ImageJ software (**b**). The electron density of the same mitochondrial or cytosolic area in TEM images was analyzed by ImageJ software, the ratio of mitochondrial or cytosolic electron density represents the relative mitochondrial mass (**c**). n=4 pieces of copper mesh for each experiment. Error bars indicate the mean ± SD of the experiments, n = 3 independent experiments, statistical significance was assessed by a two-way ANOVA. **d** HeLa cells expressing LAMP1-mCherry were treated with normoxia or hypoxia for 24h, and were stained with PKMDR (mitochondrial cristae and inner membrane stainer), and cells were then imaged by HIS-SIM. *P*-values are indicated in the figure. Source data are provided as a Source Data file.

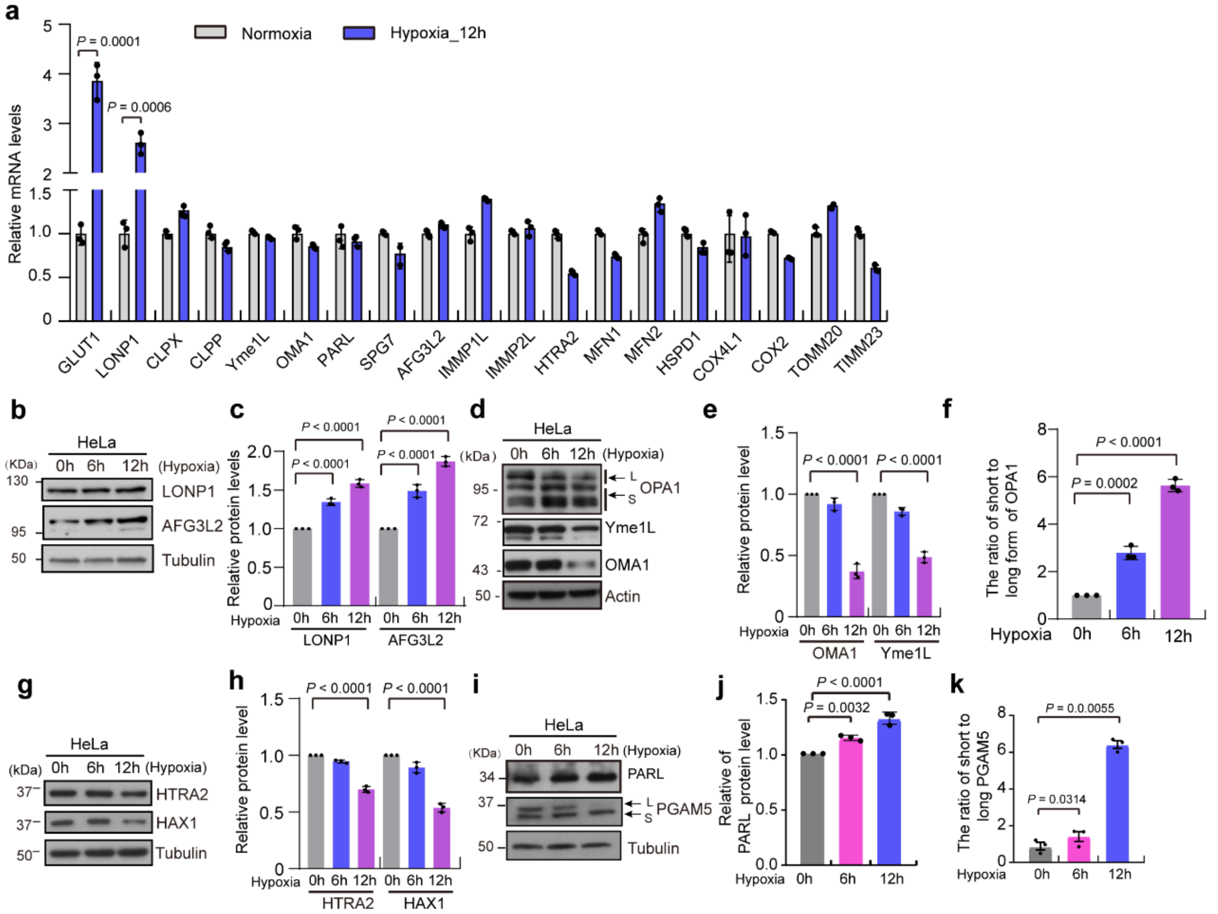

Figure S11. Mitochondrial proteases were activated under hypoxia

a HeLa cells were cultured in normoxia or hypoxia for 12h. The relative levels of the indicated mRNA in normoxia and hypoxia were analyzed by quantitative RT-PCR. Error bars indicate the mean ± SEM of the experiments, n = 3 independent experiments, statistical significance was assessed by two-tailed *t*-test. **b-k** HeLa cells were exposed to hypoxia for 0h (normoxia), 6h, and 12h. Cell lysates were analyzed by Western blotting using antibodies against LONP1 (**b**), AFG3L2 (**b**), Yme1L (**d**), OMA1 (**d**), OPA1 (**d**), HTRA2 (**g**), HAX1 (**g**), PARL (**i**), PGAM5 (**i**), Tubulin, or Actin. The relative protein level of LONP1 and AFG3L2 were further evaluated by densitometry analysis using ImageJ software (**c**). The relative protein level of OMA1 and Yme1L were further evaluated by densitometry analysis using ImageJ software (**e**). The bands of full length (long form) and the cleaved (short form) OPA1 were quantified by densitometry analysis, and the ratio of short to long form of OPA1 was further calculated (**f**). The relative protein level of HTRA2 and HAX1 were further evaluated by densitometry analysis using ImageJ software (**h**). The relative protein level of PARL were further evaluated by densitometry analysis using ImageJ software (**j**). The bands of full length (long form) and the cleaved (short form) PGAM5 were quantified by densitometry analysis, and the ratio of short to long form of

PGAM5 was further calculated (**k**). Error bars indicate the mean ± SD of the experiments, n = 3 independent experiments, statistical significance was assessed by a one-way ANOVA. *P*-values are indicated in the figure. Source data are provided as a Source Data file.

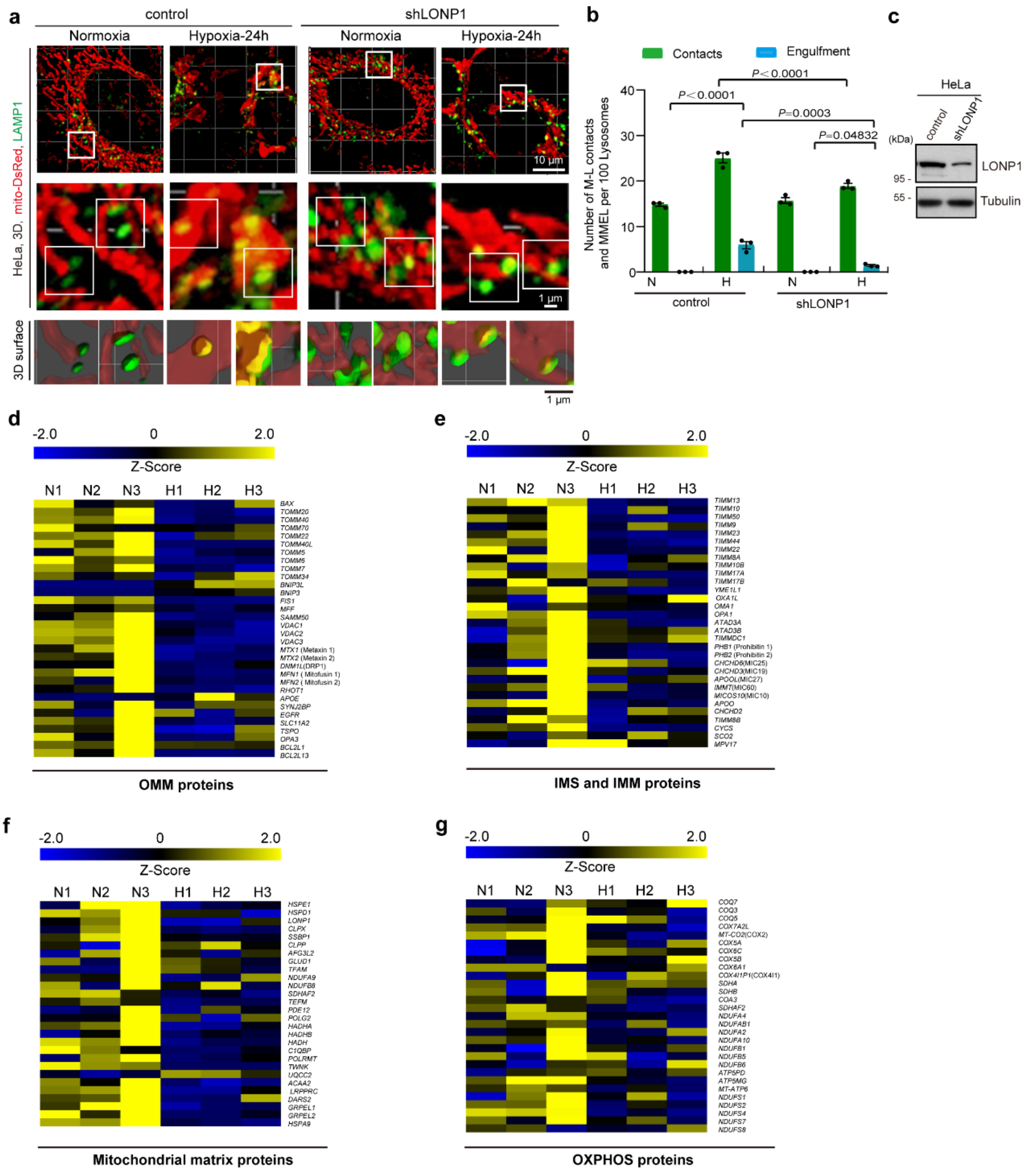

Figure S12. The effect of hypoxia on mitochondrial proteins

a-c Control, *LONP1* knockdown (sh*LONP1*) HeLa cells stably expressing mito-DsRed (mitochondria) were exposed to normoxia or hypoxia for 24h, and were immunostained with

an antibody against LAMP1 (lysosome), and analyzed by 3D imaging with confocal microscopy with Airyscan (**a**). Mitochondria (red) and lysosome (green) were displayed using 3D surface reconstructions overlaid upon original data with Imaris software. The middle images show enlargements of the boxed areas in the top images, and the bottom images are 3D surface reconstructions of the middle images. Mitochondria-lysosome contacts (contacts) and MMEL (engulfment) from 10 cells were quantified in each experiment, and the number of M-L contacts and MMEL per 100 lysosomes was displayed (**b**). Error bars indicate the mean ± SEM of the experiments, n = 3 independent experiments, statistical significance was assessed by a two-way ANOVA (**c**). Cell lysates of control or *LONP1* knockdown (sh*LONP1*) HeLa cells were analyzed by Western blotting with anti-LONP1 or anti-Tubulin antibodies. **d-g** Heat map of the relative content of mitochondrial proteins. HeLa cells were cultured in normoxia or hypoxia for 24h, and were collected for purification of mitochondria fraction, and then analyzed by mass spectrometry. The data of mass spectrometry were divided into four parts: outer mitochondrial membrane (OMM) proteins (**d**), intermembrane mitochondrial space (IMS) and inner mitochondrial membrane (IMM) proteins (**e**), mitochondrial matrix proteins (**f**), and oxidative phosphorylation (OXPHOS) complexes proteins (**g**). The data were analyzed by Z-Score, and processed for heatmap production using MeV software. "N" indicates normoxia, and "H" indicates hypoxia. *P*-values are indicated in the figure. Source data are provided as a Source Data file.

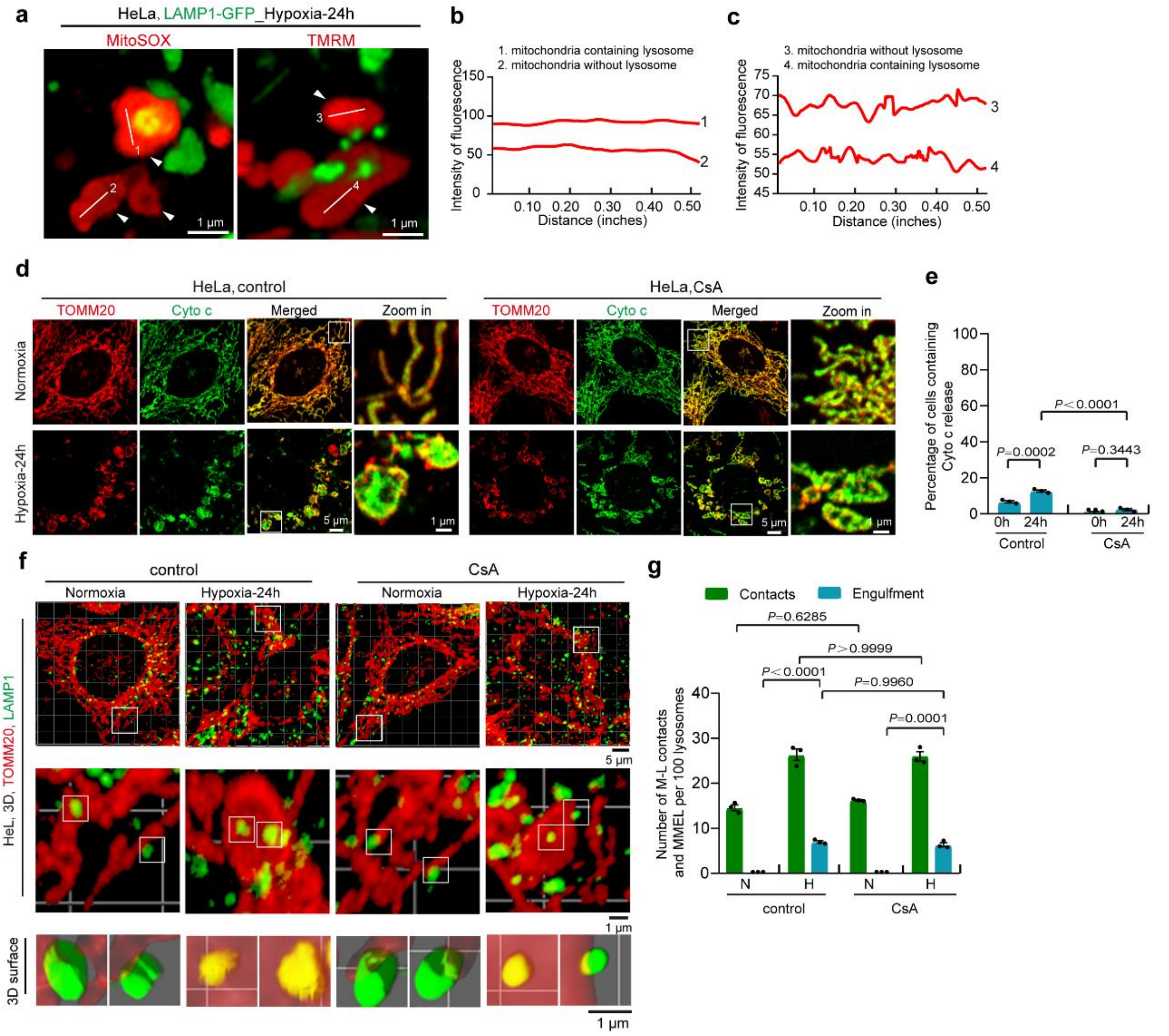

Figure S13. Megamitochondrial damage does not affect MMEL under hypoxia

a-c HeLa cells expressing LAMP1–GFP were treated with normoxia or hypoxia for 24h, and then directly stained with mitoSOX or TMRM, and analyzed and imaged by confocal microscopy (**a**). The right panels showed the fluorescence intensity of mitoSOX (**b**) or TMRM (**c**) from a line. **d-e** HeLa cells were exposed to normoxia or hypoxia in the presence of DMSO (control), or Cyclosporin A (CsA) for 24h, and were immunostained with antibodies against TOMM20 (mitochondria, red) or cytochrome c (green), and were then analyzed by confocal microscopy with Airyscan (**d**). The number of cells (n=100) containing cytochrome c release was quantified (**e**). Data with error bars are presented as mean ± SEM of the experiments, n = 3 independent experiments, statistical significance was assessed by a two-way ANOVA. **f-g** HeLa cells were treated with normoxia or hypoxia in the presence of DMSO, or Cyclosporin A (CsA) for 24h, and then immunostained with anti-LAMP1 and anti-TOMM20 antibodies, and analyzed by 3D imaging with confocal microscopy with Airyscan. Mitochondria (red) and lysosome (green) were displayed using 3D surface reconstructions overlaid upon original data

with Imaris software (**f**). The middle images show enlargements of the boxed areas in the top images, and the bottom images are 3D surface reconstructions of the middle images. The events of mitochondria-lysosome contacts (M-L contacts) and lysosome within megamitochondria (L within M) from 10 cells were then quantified (**g**) according to the criteria detailed in "Materials and Methods", and the number of "M-L contacts" and "L within M" per 100 lysosomes was displayed. Error bars indicate the mean ± SEM of the experiments, n = 3 independent experiments, statistical significance was assessed by a two-way ANOVA. *P*-values are indicated in the figure. Source data are provided as a Source Data file.
