# Supplementary material for: Hypoxia-reprogramed megamitochondrion contacts and engulfs lysosome to mediate mitochondrial self-digestion
Source: Nat Commun. 2023 Jul 11;14:4105. doi: 10.1038/s41467-023-39811-9 (PMC10336010; doi:10.1038/s41467-023-39811-9)
Supplement: Supplementary file 2 — Description of Additional Supplementary Files [file 41467_2023_39811_MOESM2_ESM.pdf]

Description of Additional Supplementary Files

File Name: Supplementary Movie 1-2
Description: HeLa cells co-expressing mito-GFP and TOMM20-mCherry were treated with normoxia (21% O_2) (supplementary video 1) or hypoxia (1% O_2) (supplementary video 2) for 24h, and then tracked by time-lapse imaging using a confocal microscope. Two videos show the processes of mitochondrial fusion under normoxic (supplementary video 1) and hypoxic (supplementary video 2) conditions, respectively.

File Name: Supplementary Movie 3
Description: HeLa cells were treated with hypoxia (1% O_2) for 24h, and were directly stained with PKMDR and were analyzed by time-lapse confocal imaging with HIS-SIM (High Sensitivity Structured Illumination Microscope). The video shows the process of mitochondrial inner membrane fusion under hypoxic conditions.

File Name: Supplementary Movie 4-5
Description: HeLa cells stably expressing mito-GFP (mitochondria) were treated with normoxia (supplementary video 4) or hypoxia (supplementary video 5) for 24h, and were directly stained with LysoTracker Red. Cells were then analyzed by 3D imaging with confocal microscopy with Airyscan. Mitochondria (green) and lysosome (red) were displayed in 3D video. White arrowhead indicates lysosome. X-axis (red), Y-axis (green), and Z-axis (blue).

File Name: Supplementary Movie 6
Description: HeLa cells stably expressing mito-GFP (mitochondria) were treated with hypoxia for 24h, and were immunostained with antibodies against LAMP1 (lysosome). Cells were then analyzed by 3D imaging with confocal microscopy with Airyscan. Mitochondria (green) and lysosome (red) were displayed in 3D video. White arrowhead indicates lysosome. X-axis (red), Y-axis (green), and Z-axis (blue).

File Name: Supplementary Movie 7-10
Description: Focused ion beam/scanning electron microscopy (FIB-SEM) FIB-SEM recording of mitochondria of HeLa cells treated with hypoxia for 12h. Displayed is an orthoslice moving through the data stack recorded by FIB-SEM, the arrows indicate "the lysosome within mitochondrion" (supplementary video 7) or "the mitochondrion engulfing lysosome" (supplementary video 9). Then, FIB-SEM images of several segments of mitochondrion and lysosome from HeLa cells were used for 3D reconstruction by the 3D IMOD software, and "the lysosome within mitochondrion" (supplementary video 8) or "the mitochondrion engulfing lysosome" (supplementary video 10) were displayed. Outer mitochondrial membrane (*white*), inner mitochondrial membrane (*cyan*), and lysosomal membrane (*Purple- red)*.

File Name: Supplementary Movie 11
Description: Living HeLa cells expressing CTSB–mCherry (lysosomal enzymes) and

mito-GFP (mitochondria) were treated with hypoxia (1% O_2) for 24h, and then were tracked and imaged with confocal microscopy with Airyscan. The video shows the changes of lysosomal enzymes in mitochondria under hypoxic conditions.
