# Supplementary material for: Hypoxia-reprogramed megamitochondrion contacts and engulfs lysosome to mediate mitochondrial self-digestion
Source: Nat Commun. 2023 Jul 11;14:4105. doi: 10.1038/s41467-023-39811-9 (PMC10336010; doi:10.1038/s41467-023-39811-9)
Supplement: Supplementary file 14 — Reporting Summary [file 41467_2023_39811_MOESM14_ESM.pdf]

nature portfolio

Corresponding author(s): Zhiyin Song

Last updated by author(s): Mar 14, 2023

Reporting Summary

Nature Portfolio wishes to improve the reproducibility of the work that we publish. This form provides structure for consistency and transparency in reporting. For further information on Nature Portfolio policies, see our Editorial Policies and the Editorial Policy Checklist.

Statistics

For all statistical analyses, confirm that the following items are present in the figure legend, table legend, main text, or Methods section.

n/a	Confirmed	
☐	☒	The exact sample size (*n*) for each experimental group/condition, given as a discrete number and unit of measurement
☐	☒	A statement on whether measurements were taken from distinct samples or whether the same sample was measured repeatedly
☐	☒	The statistical test(s) used AND whether they are one- or two-sided<br>*Only common tests should be described solely by name; describe more complex techniques in the Methods section.*
☒	☐	A description of all covariates tested
☒	☐	A description of any assumptions or corrections, such as tests of normality and adjustment for multiple comparisons
☐	☒	A full description of the statistical parameters including central tendency (e.g. means) or other basic estimates (e.g. regression coefficient) AND variation (e.g. standard deviation) or associated estimates of uncertainty (e.g. confidence intervals)
☐	☒	For null hypothesis testing, the test statistic (e.g. *F*, *t*, *r*) with confidence intervals, effect sizes, degrees of freedom and *P* value noted<br>*Give P values as exact values whenever suitable.*
☒	☐	For Bayesian analysis, information on the choice of priors and Markov chain Monte Carlo settings
☒	☐	For hierarchical and complex designs, identification of the appropriate level for tests and full reporting of outcomes
☒	☐	Estimates of effect sizes (e.g. Cohen's *d*, Pearson's *r*), indicating how they were calculated

Our web collection on statistics for biologists contains articles on many of the points above.

Software and code

Policy information about availability of computer code

Data collection

Images , Three-dimensional (3D) confocal imaging and Time-lapse movies were collected using ZEISS LSM880 laser scanning confocal microscope controlled by Zeiss Zen software (version 16.02.306) equipped with Airyscan module and Leica SP8 controlled by LAS X software(version 3.3, Leica and version LAS4.13). Proteomics data was collected on Liquid Chromatography system coupled to a Q Exactive HF Orbitrap and an EASYnLC 1000 ultra-high-pressure system via anano-electrospray ionsource (Thermo Fisher Scientific). The images were collected a JEM-1400Plus (JEOL) transmission electron microscope at an acceleration voltage of 100 kV. And then Focused ion beam milling and SEM imaging were carried out with an FEI Helios NanoLab G3 UC (from Tsinghua, China). Target mRNA expression were used the iCycler real-time PCR Detection System (Bio-Rad). The concentration of mitochondrial proteins was measured by the Bradford assay(Bio-Rad). All SIM images were analyzed using the Hessian-SIM microscopy .The images of High Pressure Freezing and immunoelectron microscopy samples were collected a JEM-1400Plus (JEOL) transmission electron microscope at an acceleration voltage of 100 kV. OMA1 knockout vectors with Yme1L were obtained by LentiCRISPR (V2).

Data analysis

3D surface reconstructions overlaid upon original data were analyzed and produced by Imaris software(Imaris v9.6, Oxford Instruments).
Statistical analyses were performed using GraphPad Prism 8.0 (Graphpad Prism 8.2.1)and Microsoft Excel (v16.52, Office 2013).
The selected mitochondrial images were there-dimensional (3D) reconstituted by Amira software, and the mitochondrial 3D presentation was rendered by IMOD software(version 5.5.1).
Ratiometric (552 nm: 448 nm) analysis of Mito-Keima mitophagy assay was performed using ImageJ software(v2.3.0/1.53f).
Mass Spectrometry Data were analyzed by Z-Score, and processed for heatmap production using MeV software(version 4.6.0).
The fold change of target mRNA expression was calculated using the 2-ΔΔCT method.
The fluorescence intensity of Mitochondrial ROS Measurement was analyzed by ImageJ software(v2.3.0/1.53f).
The fluorescence intensity of Mitochondrial TMRM Measurement was analyzed by ImageJ software(v2.3.0/1.53f).
The fluorescence intensity of lysosomal enzyme within megamitochondria Measurement was analyzed by ImageJ software(v2.3.0/1.53f).

The relative protein levels were evaluated by densitometry analysis using ImageJ software(v2.3.0/1.53f).
The electron density of the same mitochondrial or cytosolic area in TEM images was analyzed by ImageJ software(v2.3.0/1.53f).

For manuscripts utilizing custom algorithms or software that are central to the research but not yet described in published literature, software must be made available to editors and reviewers. We strongly encourage code deposition in a community repository (e.g. GitHub). See the Nature Portfolio guidelines for submitting code & software for further information.

Data

Policy information about availability of data

All manuscripts must include a data availability statement. This statement should provide the following information, where applicable:

- Accession codes, unique identifiers, or web links for publicly available datasets
- A description of any restrictions on data availability
- For clinical datasets or third party data, please ensure that the statement adheres to our policy

All the data and relevant materials, including reagents and primers, supporting the conclusions included in the manuscript are available within the paper and its supplementary information. Source data are provided with this paper. The source data underlying Supplementary Figs 12 D,E, F& G are provided as a Source Data file.(The raw data of the MSMS was deposited at the WEB Link).

Human research participants

Policy information about studies involving human research participants and Sex and Gender in Research.

Reporting on sex and gender	Our research content dose not cover this field work.
Population characteristics	Our research content dose not cover this field work.
Recruitment	Our research content dose not cover this field work.
Ethics oversight	Our research content dose not cover this field work.

Note that full information on the approval of the study protocol must also be provided in the manuscript.

Field-specific reporting

Please select the one below that is the best fit for your research. If you are not sure, read the appropriate sections before making your selection.

☒ Life sciences ☐ Behavioural & social sciences ☐ Ecological, evolutionary & environmental sciences

For a reference copy of the document with all sections, see nature.com/documents/nr-reporting-summary-flat.pdf

Life sciences study design

All studies must disclose on these points even when the disclosure is negative.

Sample size	For most in vitro experiments, at least 3 independent samples were used for each experimental group. This gives enough power to detect the differences. Due to practicalities, the minimum sample size for mass spectrometry was limited to 3. considering the labor, time and cost of sample generation and analysis. As noted in the manuscript, multiple comparison correction was used to detect differential expression of mitochondria-associated proteins under hypoxic conditions by statistical comparison.
Data exclusions	No data were excluded from the analyses.
Replication	All of experiments were performed at least three times. Experimental results are reliably reproduced.
Randomization	The experiments with cell samples were allocated into experimental groups with different treatment, including siRNAs or drugs or hyporxia.
Blinding	For quantification of extent of mitochondria-lysosome contacts or MMEL [all lysosomes (more than 500) , a blinded quantification was applied. 3D confocal imaging with ZEISS LSM880 microscope with Airyscan was then performed, and 3D images were analyzed and used for the quantification of mitochondria-lysosome contacts or MMEL were used for quantification in each experiment, at least 3 independent experiments]. The data were then analyzed, and the number of mitochondria-lysosome contacts or MMEL per 100 lysosomes was shown ("per 10 cells" or "per 100 mitochondria" was not used because the number of lysosomes is significantly increased and mitochondrial morphology is dramatically changed in each cell upon hypoxia). Colocalization of lysosome with mitochondria was analyzed by ImageJ software, > 5% and ≤50% of a lysosome colocalizing with mitochondrion was scored into "mitochondria-lysosome contacts", and >50% of a lysosome colocalizing with mitochondrion was scored into "MMEL". At least 3 independent experiments were performed for the quantification of MMEL. Statistical analysis was performed using Prism 8 (GraphPad Software). In all electron microscopy or confocal photographic images, researchers were blinded to the assignment of mitochondrial and lysosomal interactions in groups. In vitro experiments Conducted by one person/experiment, so blinding is not possible.

Behavioural & social sciences study design

All studies must disclose on these points even when the disclosure is negative.

Study description	Our research content dose not cover this field work.
Research sample	Our research content dose not cover this field work.
Sampling strategy	Our research content dose not cover this field work.
Data collection	Our research content dose not cover this field work.
Timing	Our research content dose not cover this field work.
Data exclusions	Our research content dose not cover this field work.
Non-participation	Our research content dose not cover this field work.
Randomization	Our research content dose not cover this field work.

Ecological, evolutionary & environmental sciences study design

All studies must disclose on these points even when the disclosure is negative.

Study description	Our research content dose not cover this field work.
Research sample	Our research content dose not cover this field work.
Sampling strategy	Our research content dose not cover this field work.
Data collection	Our research content dose not cover this field work.
Timing and spatial scale	Our research content dose not cover this field work.
Data exclusions	Our research content dose not cover this field work.
Reproducibility	Our research content dose not cover this field work.
Randomization	Our research content dose not cover this field work.
Blinding	Our research content dose not cover this field work.

Did the study involve field work? ☐ Yes ☒ No

Reporting for specific materials, systems and methods

We require information from authors about some types of materials, experimental systems and methods used in many studies. Here, indicate whether each material, system or method listed is relevant to your study. If you are not sure if a list item applies to your research, read the appropriate section before selecting a response.

Materials & experimental systems

n/a	Involved in the study	
☐	☒	Antibodies
☐	☒	Eukaryotic cell lines
☒	☐	Palaeontology and archaeology
☒	☐	Animals and other organisms
☒	☐	Clinical data
☒	☐	Dual use research of concern

Methods

n/a	Involved in the study	
☒	☐	ChIP-seq
☒	☐	Flow cytometry
☒	☐	MRI-based neuroimaging

Antibodies

Antibodies used

Antibody (Supplier, Cat. No) Dilution factor
Anti-TOM20 (Proteintech, 11802-1-AP) 1 : 5000, anti-LAMP1 (SantaCruz, sc-20011) 1 : 2500, anti-TIM23 (BD Biosciences,611222) 1 : 3500, anti-COX2 (Abcam, ab15191) 1 : 2500, anti-HSP60 (SantaCruz, sc-13115) 1 : 5000, anti-LONP1 (Proteintech, 15440-1-AP) 1 : 2500, anti-COX4(Proteintech, 11242-1-AP) 1 : 2500, anti-Drp1 (BD Biosciences, 611738) 1 : 2000, anti-Mff (SantaCruz, sc-398617) 1 : 2000, anti-CSTD (ABclonal, a13292) 1 : 2000, anti-ATG5 (ABclonal, a0203) 1 : 2000, anti-Mid49 (Proteintech, 16413-1-AP) 1 : 2500, anti-AFG3L2 (ABclonal, A15393) 1 : 2000, anti-OPA1 (BD Biosciences, 612607) 1 : 1000, anti-HIF-1α (BD Biosciences, 610909) 1 : 800, anti-GFP (SantaCruz, sc-9996) 1 : 3000, anti-Yme1L (Proteintech, 11510-1-AP) 1 : 2000, anti-OMA1 (SantaCruz, H-11) 1 : 2000, anti-PARL (Proteintech, 26679-1-AP) 1 : 2000, anti-LAMP2 (Servicebio, GB11330) 1 : 2000, anti-Fis1 (Proteintech, 10956-1-AP) 1 : 2000, anti-Syntaxin17(Proteintech, 17815-1-AP) 1 : 2000, anti-HAX-1 (Proteintech, 11266-1-AP) 1 : 2000, anti-HTRA2 (Proteintech, 15775-1-AP) 1 : 2000, anti-SNAP29 (ABclonal, A4290) 1 : 2000, anti-VAMP7 (Proteintech, 22268-1-AP) 1 : 2000, anti-Actin (GNI, GNI4110-BA) 1 : 5000, anti-Tubulin (GNI, GNI4110-BT) 1 : 5000. anti-PGAM5 was a gift from Quan Chen 1 : 2000. HRP-conjugated secondary antibodies were from Jackson ImmunoResearch Laboratories (31160) 1 : 400, donkey anti-mouse IgG Alexa Fluor-647 (Jackson, 715–605-150) 1 : 400, donkey anti-mouse IgG Alexa Fluor-488 (Jackson) 1 : 400, goat anti-rabbit IgG CY3 (Jackson, 111–165-003) 1 : 400, goat anti-mouse IgG Alexa Fluor-594(Yeasen,33212ES60) 1 : 400, Cyclosporin A(MedChemExpress,5865-13-3).

Validation

Antibody (Supplier, Cat. No) Dilution factor/Validation
Anti-TOM20 (Proteintech, 11802-1-AP) 1 : 5000/Manufacturer COA detected TOM20 in HEK-293 cells and HeLa cells (Western and microscopy) , anti-LAMP1 (SantaCruz, sc-20011) 1 : 2500/Manufacturer COA detected LAMP1 in U-937 cells and ECV304 cells (Western) , anti-TIM23 (BD Biosciences,611222) 1 : 3500/Manufacturer COA detected TIM23 in RSV-3T3 cells (Western) , anti-COX2 (Abcam, ab15191) 1 : 2500/Manufacturer COA detected COX2 in B16-F10 cells and Mouse retina tissue (Western) , anti-HSP60 (SantaCruz, sc-13115) 1 : 5000/Manufacturer COA detected HSP60 in HEK-293 cells (Western), anti-LONP1 (Proteintech, 15440-1-AP) 1 : 2500/Manufacturer COA detected LONP1 in mouse heart tissue and HeLa cells (Western) , anti-COX4(Proteintech, 11242-1-AP) 1 : 2500/Manufacturer COA detected COX4 in HepG2 cells and HeLa cells (Western and microscopy) , anti-Drp1 (BD Biosciences, 611738) 1 : 2000/Manufacturer COA detected Drp1 in HeLa cells (Western) , anti-Mff (SantaCruz, sc-398617) 1 : 2000/Manufacturer COA detected Mff in 293T cells and HeLa cells (Western and microscopy) , anti-CSTD (ABclonal, a13292) 1 : 2000/Manufacturer COA detected CSTD in SK-BR-3 cells (Western) , anti-ATG5 (ABclonal, a0203) 1 : 2000/Manufacturer COA detected ATG5 in HepG2 cells and SH-SY5Y cells (Western) , anti-Mid49 (Proteintech, 16413-1-AP) 1 : 2500/Manufacturer COA detected Mid49 in HepG2 cells and HEK-293 cells (Western) , , anti-AFG3L2 (ABclonal, A15393) 1 : 2000/Manufacturer COA detected AFG3L2 in HepG2 cells and HeLa cells (Western), anti-OPA1 (BD Biosciences, 612607) 1 : 1000/Manufacturer COA detected OPA1 in K-562 cells (Western), anti-HIF-1α (BD Biosciences, 610909) 1 : 800/Manufacturer COA detected HIF-1α in HeLa cells (Western), anti-GFP (SantaCruz, sc-9996) 1 : 3000/Manufacturer COA detected GFP in COS cells transfected with GFP fusion protein (Westernand microscopy), anti-Yme1L (Proteintech, 11510-1-AP) 1 : 2000/Manufacturer COA detected Yme1L in HepG2 cells and HeLa cells (Western), anti-OMA1 (SantaCruz, H-11) 1 : 2000/Manufacturer COA detected OMA1 in 293T cells and HeLa cells (Western) , anti-PARL (Proteintech, 26679-1-AP) 1 : 2000/Manufacturer COA detected PARL in 293T cells and HeLa cells (Western),, anti-LAMP2 (Servicebio, GB11330) 1 : 2000/Manufacturer COA detected LAMP2 in 293T cells and HeLa cells (Western), anti-Fis1 (Proteintech, 10956-1-AP) 1 : 2000/ Manufacturer COA detected Fis1 in 293T cells and HeLa cells (Western), anti-Syntaxin17(Proteintech, 17815-1-AP) 1 : 2000/ Manufacturer COA detected Syntaxin17 in HpG2 cells (Western), anti-HAX-1 (Proteintech, 11266-1-AP) 1 : 2000/Manufacturer COA detected HAX-1 in HeLa cells (Western), anti-HTRA2 (Proteintech, 15775-1-AP) 1 : 2000/Manufacturer COA detected HTRA2 in 293T cells and HeLa cells (Western), anti-SNAP29 (ABclonal, A4290) 1 : 2000/Manufacturer COA detected SNAP29 in HeLa cells (Western), anti-VAMP7 (Proteintech, 22268-1-AP) 1 : 2000/Manufacturer COA detected VAMP7 in mouse brain tissue (Western), anti-Actin (GNI, GNI4110-BA) 1 : 5000/Manufacturer COA detected Actin in HeLa cells (Western), anti-Tubulin (GNI, GNI4110-BT) 1 : 5000/ Manufacturer COA detected Tubulin in HeLa cells (Western). anti-PGAM5 was a gift from Quan Chen 1 : 2000. HRP-conjugated secondary antibodies were from Jackson ImmunoResearch Laboratories (31160) 1 : 400, donkey anti-mouse IgG Alexa Fluor-647 (Jackson, 715–605-150) 1 : 400, donkey anti-mouse IgG Alexa Fluor-488 (Jackson) 1 : 400, goat anti-rabbit IgG CY3 (Jackson, 111–165-003) 1 : 400, goat anti-mouse IgG Alexa Fluor-594(Yeasen,33212ES60) 1 : 400, Cyclosporin A(MedChemExpress,5865-13-3).

Eukaryotic cell lines

Policy information about cell lines and Sex and Gender in Research

Cell line source(s)

Human cervical cancer HeLa cell line (Dr. XiaojunXia, SUN YAT-SEN UNIVERSITY), Human colorectal carcinoma HCT116 cell line (Dr. XiaojunXia, SUN YAT-SEN UNIVERSITY), Human breast cancer MCF-7 cell line (Dr. XiaojunXia, SUN YAT-SEN UNIVERSITY) and Human embryonic kidney 293T cells (Dr. XiaojunXia, SUN YAT-SEN UNIVERSITY),Mouse Embryonic Fibroblasts (MEFs) cell line (Dr. Zhiyin Song(Corresponding author)) were grown in DMEM (Invitrogen) supplemented with 10% fetal bovine serum (Gibco) and 1% penicillin/streptomycin.

Authentication

All cell lines used in this study was authenticated by the provider.

Mycoplasma contamination

All of the cell lines used in this study are free of mycoplasma contamination.

Commonly misidentified lines
(See ICLAC register)

No commonly misidentified cell lines were used.
